# Genetic Diversity of Polymyxin Resistance Genes in *Klebsiella pneumoniae* Clinical Isolates

**DOI:** 10.1111/mec.70234

**Published:** 2026-01-20

**Authors:** Daniel Miceli Serwy, Maria Eduarda Rocha Conde, Ana Luiza Carneiro Alencar, Roberto Leonan Morim Novaes, Josué da Costa Lima‐Junior, Fabio Faria da Mota, Ana Paula Carvalho‐Assef, Teca Calcagno Galvao, Viviane Zahner

**Affiliations:** ^1^ Fiocruz, Laboratório de Bacteriologia Aplicada a Saúde Única e Resistência Antimicrobiana Instituto Oswaldo Cruz Rio de Janeiro RJ Brazil; ^2^ Fiocruz, Laboratório de Imunoparasitologia Instituto Oswaldo Cruz Rio de Janeiro RJ Brazil; ^3^ Fiocruz Mata Atlântica Rio de Janeiro RJ Brazil; ^4^ Fiocruz, Laboratório de Biologia Computacional e Sistemas Instituto Oswaldo Cruz Rio de Janeiro RJ Brazil; ^5^ Fiocruz, Laboratório Integrado ‐ Simulídeos e Oncocercose & Entomologia Médica e Forense Instituto Oswaldo Cruz Rio de Janeiro RJ Brazil

**Keywords:** polygenic adaption, polymyxin resistance, population genetics, positive selection, resistance evolution, two component systems

## Abstract

This study investigates the genetic diversity and evolutionary mechanisms driving polymyxin resistance in *Klebsiella pneumoniae* , a critical priority pathogen. By analysing *mgrB*, *phoPQ* and *pmrAB* genes in susceptible (PM‐S) and resistant (PM‐R) populations through neutrality tests (Tajima *D*, Fu & Li's *D*) we uncovered polygenic adaptation and positive selection as a key driver of resistance. High genetic diversity was observed across all *loci*, with *mgrB* insertions dominating PM‐R populations. Negative Tajima and Fu & Li's *D* values and excess rare alleles revealed recent population expansions linked to the reintroduction of polymyxins in the 2010s. Positive selection via selective sweeps was detected in PM‐R isolates, exemplified by the rapid spread of haplotype 27, which presents *mgrB* insertions, the major determinant of LPS modification pathway hyperactivation. The expansion of this haplotype suggests that horizontal gene transfer accelerates resistance dissemination. The elevated genetic diversity observed in the *phoPQ* and *pmrAB* systems among isolates harbouring *mgrB* alterations may reflect reduced adaptive fitness costs, enabling the preservation of genomic variability despite sustained selective pressures. Our results demonstrate that polymyxin resistance arises through polygenic adaptation and positive selection, combining *de novo* mutations, recombination and selection‐driven sweeps. These dynamics threaten to exacerbate resistance in hospital environments, emphasising the need for genomic surveillance and alternative therapies. This study bridges molecular evolution and clinical epidemiology, offering insights into the resilience of *K. pneumoniae* and the ecological drivers of antimicrobial resistance.

## 1 Introduction

Antimicrobial resistance (AMR) is a naturally occurring phenomenon characterised by the ability of microorganisms to withstand the effects of antimicrobial drugs. First noted by Fleming following the discovery of penicillin (Fleming 1945), its medical significance escalated with the emergence of penicillin resistance, a trend exacerbated by the widespread use of antibiotics during World War II (Podolsky 2015). Despite continuous efforts by the pharmaceutical industry to develop new antimicrobial agents, the evolution of resistance has persistently outpaced drug innovation (Ventola 2015). Today, AMR is a global crisis, with environmental dissemination facilitated by multiple vectors, including agricultural runoff, hospital effluents, domestic wastewater and aquaculture systems, underscoring its status as a One Health challenge (Hernando‐Amado et al. 2019).

Projections indicate that by 2050, AMR‐related deaths could rival annual cancer mortality rates, signalling an ongoing ‘silent pandemic’ (O'Neill 2016). In response, the World Health Organization (WHO) established a Global Priority Pathogens List in 2017, updated in 2024, to prioritise research and surveillance of critical threats (WHO 2024). Among these, carbapenem‐resistant Enterobacterales (CRE) are classified as urgent, with carbapenem‐resistant *Klebsiella pneumoniae* (CRKP) posing a paramount risk (WHO 2024). Carbapenems, a type of β‐lactam antibiotics reserved as last‐line therapies for nosocomial infections, are increasingly rendered ineffective by CRKP (Nordmann et al. 2011).

*K. pneumoniae* , a Gram‐negative bacterium, emerged as a clinically significant pathogen in the 1970s due to its propensity for multidrug resistance (MDR) and role in hospital‐acquired infections (Podschun and Ullmann 1998). By the 1980s, extended‐spectrum β‐lactamase (ESBL)‐producing strains resistant to cephalosporins were documented (Paterson and Bonomo 2005). The early 2000s marked the rise of carbapenemase KPC producers, which remain a global healthcare burden (Munoz‐Price et al. 2013).

CRKP's resilience stems from its ability to acquire and transmit mobile genetic elements (MGEs), enabling horizontal gene transfer (HGT) within and across species boundaries (Partridge et al. 2018). This genetic plasticity, coupled with resistance to last‐line β‐lactams and third‐generation cephalosporins, has necessitated alternative therapies, such as polymyxins (Nation et al. 2014). Polymyxins, cationic peptides, target the anionic lipopolysaccharide (LPS) of *K. pneumoniae* , disrupting membrane integrity and inducing cell lysis (Velkov et al. 2013). However, escalating polymyxin use has driven the emergence and dissemination of resistant strains, mediated by mutations in genes such as *mgrB*, *phoPQ*, *pmrAB* and MGE‐borne *mcr* variants (Moffatt et al. 2010). These genetic alterations modify lipid A via phosphoethanolamine or L‐arabinose additions, reducing polymyxin binding affinity (Olaitan et al. 2014).

While prior studies have focused on resistance‐associated mutations and clonal lineages (Poirel, Jayol, and Nordmann 2015), emerging evidence suggests polymyxin resistance is multifactorial, influenced by environmental conditions and infection site dynamics (El‐Sayed Ahmed et al. 2020). Notably, genetic diversity in susceptible strains and the evolutionary trajectories of resistance genes remain underexplored, with limited application of population genetics to elucidate allele frequency shifts, spatiotemporal evolution or selective pressures in *K. pneumoniae* populations (Wyres and Holt 2018).

This study aims to delineate the evolutionary forces shaping five key polymyxin resistance determinants (*mgrB*, *phoP*, *phoQ*, *pmrA*, *pmrB*) in *K. pneumoniae* . By analysing genetic diversity in both susceptible and resistant strains under varying polymyxin concentrations, we seek to unravel how antimicrobial pressure drives selection in different *K. pneumoniae* subpopulations.

## 2 Materials and Methods

### 2.1 Study Design

The study sample consisted of isolates for which there were polymyxin susceptibility test results and sequence information for the five genes of interest (*pmrAB*, *phoPQ* and *mgrB*), allowing analysis of genetic diversity in relation to the susceptibility phenotype. Isolates were sampled from three sources. Twenty‐one isolates were processed and analysed for the current study (SisGen registration numbers AD6C6D8, AF42316 and A5F86E8). One hundred and forty‐five isolates for which sequence and polymyxin susceptibility profiles were available in Genbank. Finally, forty‐eight isolates previously described (Conceição Neto et al. 2022), yielding a total of 214 samples (Table 1). Given the importance of following gold standards protocols, isolates included in the study had their polymyxin B or E (colistin) Minimum Inhibitory Concentration (MIC) values determined using broth microdilution assays (207 isolates), adhering to standardised protocols (BrCAST 2023; CLSI 2023; EUCAST 2023). The MIC of six isolates was determined by *E*‐tests, and for one isolate there was no description of the method used. For population and evolutionary analyses, isolates were stratified into two groups: polymyxin‐susceptible and polymyxin‐resistant strains. The primary criteria for classification was the MIC, with values exceeding 2 mg/L indicating a resistant phenotype, consistent with international breakpoint guidelines (CLSI 2023; EUCAST 2023).

**TABLE 1:** Isolates included in the study.

Strain	MIC	Phenotype	Susceptibility test	Source and reference	Country	Polymyxin	Year of collection
129	32	Resistant	Broth microdilution (BrCAST)	Sanger (This Work)	Brazil	B	2023
153	16	Resistant	Broth microdilution (BrCAST)	Sanger (This Work)	Brazil	B	2023
154	16	Resistant	Broth microdilution (BrCAST)	Sanger (This Work)	Brazil	B	2023
155	16	Resistant	Broth microdilution (BrCAST)	Sanger (This Work)	Brazil	B	2023
201	8	Resistant	Broth microdilution (BrCAST)	Sanger (This Work)	Brazil	B	2023
219	16	Resistant	Broth microdilution (BrCAST)	Sanger (This Work)	Brazil	B	2023
249	0.5	Susceptible	Broth microdilution (BrCAST)	Sanger (This Work)	Brazil	B	2023
257	64	Resistant	Broth microdilution (BrCAST)	Sanger (This Work)	Brazil	B	2023
417	64	Resistant	Broth microdilution (BrCAST)	Sanger (This Work)	Brazil	B	2023
446	64	Resistant	Broth microdilution (BrCAST)	Sanger (This Work)	Brazil	B	2023
7559	16	Resistant	Broth microdilution (BrCAST)	Sanger (This Work)	Brazil	B	2023
7585	1	Susceptible	Broth microdilution (BrCAST)	Sanger (This Work)	Brazil	B	2023
CCBH22128	64	Resistant	Broth microdilution (BrCAST)	Sanger (Conceição‐Neto et al. 2022)	Brazil	B	2016
CCBH22137	16	Resistant	Broth microdilution (BrCAST)	Sanger (Conceição‐Neto et al. 2022)	Brazil	B	2016
CCBH22143	32	Resistant	Broth microdilution (BrCAST)	Sanger (Conceição‐Neto et al. 2022)	Brazil	B	2016
CCBH22206	64	Resistant	Broth microdilution (BrCAST)	Sanger (Conceição‐Neto et al. 2022)	Brazil	B	2016
CCBH22237	32	Resistant	Broth microdilution (BrCAST)	Sanger (Conceição‐Neto et al. 2022)	Brazil	B	2016
CCBH22240	32	Resistant	Broth microdilution (BrCAST)	Sanger (Conceição‐Neto et al. 2022)	Brazil	B	2016
CCBH22382	64	Resistant	Broth microdilution (BrCAST)	Sanger (Conceição‐Neto et al. 2022)	Brazil	B	2016
CCBH22391	32	Resistant	Broth microdilution (BrCAST)	Sanger (Conceição‐Neto et al. 2022)	Brazil	B	2016
CCBH22397	64	Resistant	Broth microdilution (BrCAST)	Sanger (Conceição‐Neto et al. 2022)	Brazil	B	2016
CCBH22399	64	Resistant	Broth microdilution (BrCAST)	Sanger (Conceição‐Neto et al. 2022)	Brazil	B	2016
CCBH22404	4	Resistant	Broth microdilution (BrCAST)	Sanger (Conceição‐Neto et al. 2022)	Brazil	B	2016
CCBH22408	16	Resistant	Broth microdilution (BrCAST)	Sanger (Conceição‐Neto et al. 2022)	Brazil	B	2016
CCBH22462	16	Resistant	Broth microdilution (BrCAST)	Sanger (Conceição‐Neto et al. 2022)	Brazil	B	2016
CCBH22466	64	Resistant	Broth microdilution (BrCAST)	Sanger (Conceição‐Neto et al. 2022)	Brazil	B	2016
CCBH22481	64	Resistant	Broth microdilution (BrCAST)	Sanger (Conceição‐Neto et al. 2022)	Brazil	B	2016
CCBH22491	32	Resistant	Broth microdilution (BrCAST)	Sanger (Conceição‐Neto et al. 2022)	Brazil	B	2016
CCBH22609	64	Resistant	Broth microdilution (Brcast)	Sanger (Conceição‐Neto et al. 2022)	Brazil	B	2016
CCBH22625	64	Resistant	Broth microdilution (Brcast)	Sanger (Conceição‐Neto et al. 2022)	Brazil	B	2016
CCBH22653	128	Resistant	Broth microdilution (BrCAST)	Sanger (Conceição‐Neto et al. 2022)	Brazil	B	2016
CCBH22675	64	Resistant	Broth microdilution (BrCAST)	Sanger (Conceição‐Neto et al. 2022)	Brazil	B	2016
CCBH22740	64	Resistant	Broth microdilution (BrCAST)	Sanger (Conceição‐Neto et al. 2022)	Brazil	B	2016
CCBH22997	16	Resistant	Broth microdilution (BrCAST)	Sanger (Conceição‐Neto et al. 2022)	Brazil	B	2016
CCBH22999	32	Resistant	Broth microdilution (BrCAST)	Sanger (Conceição‐Neto et al. 2022)	Brazil	B	2016
CCBH23000	16	Resistant	Broth microdilution (BrCAST)	Sanger (Conceição‐Neto et al. 2022)	Brazil	B	2016
CCBH23001	32	Resistant	Broth microdilution (BrCAST)	Sanger (Conceição‐Neto et al. 2022)	Brazil	B	2016
CCBH23024	32	Resistant	Broth microdilution (BrCAST)	Sanger (Conceição‐Neto et al. 2022)	Brazil	B	2016
CCBH23031	64	Resistant	Broth microdilution (Brcast)	Sanger (Conceição‐Neto et al. 2022)	Brazil	B	2016
CCBH23043	32	Resistant	Broth microdilution (BrCAST)	Sanger (Conceição‐Neto et al. 2022)	Brazil	B	2016
CCBH23048	32	Resistant	Broth microdilution (BrCAST)	Sanger (Conceição‐Neto et al. 2022)	Brazil	B	2016
CCBH23050	32	Resistant	Broth microdilution (BrCAST)	Sanger (Conceição‐Neto et al. 2022)	Brazil	B	2016
CCBH23064	128	Resistant	Broth microdilution (BrCAST)	Sanger (Conceição‐Neto et al. 2022)	Brazil	B	2016
CCBH23097	64	Resistant	Broth microdilution (BrCAST)	Sanger (Conceição‐Neto et al. 2022)	Brazil	B	2016
CCBH23167	16	Resistant	Broth microdilution (BrCAST)	Sanger (Conceição‐Neto et al. 2022)	Brazil	B	2016
CCBH23171	32	Resistant	Broth microdilution (BrCAST)	Sanger (Conceição‐Neto et al. 2022)	Brazil	B	2016
CCBH23220	128	Resistant	Broth microdilution (BrCAST)	Sanger (Conceição‐Neto et al. 2022)	Brazil	B	2016
CCBH23247	32	Resistant	Broth microdilution (BrCAST)	Sanger (Conceição‐Neto et al. 2022)	Brazil	B	2016
CCBH23296	64	Resistant	Broth microdilution (BrCAST)	Sanger (Conceição‐Neto et al. 2022)	Brazil	B	2016
CCBH23323	128	Resistant	Broth microdilution (BrCAST)	Sanger (Conceição‐Neto et al. 2022)	Brazil	B	2016
CCBH23368	4	Resistant	Broth microdilution (BrCAST)	Sanger (Conceição‐Neto et al. 2022)	Brazil	B	2016
CCBH23454	16	Resistant	Broth microdilution (BrCAST)	Sanger (Conceição‐Neto et al. 2022)	Brazil	B	2016
CCBH23615	128	Resistant	Broth microdilution (BrCAST)	Sanger (Conceição‐Neto et al. 2022)	Brazil	B	2016
CCBH23650	64	Resistant	Broth microdilution (BrCAST)	Sanger (Conceição‐Neto et al. 2022)	Brazil	B	2016
CCBH23661	128	Resistant	Broth microdilution (BrCAST)	Sanger (Conceição‐Neto et al. 2022)	Brazil	B	2016
CCBH23663	128	Resistant	Broth microdilution (BrCAST)	Sanger (Conceição‐Neto et al. 2022)	Brazil	B	2016
CCBH23741	128	Resistant	Broth microdilution (BrCAST)	Sanger (Conceição‐Neto et al. 2022)	Brazil	B	2016
CCBH33272	2	Susceptible	Broth microdilution (Brcast)	Sanger (This Work)	Brazil	B	2023
CCBH72480	2	Susceptible	Broth microdilution (BrVCAST)	Sanger (Conceição‐Neto et al. 2022)	Brazil	B	2016
CCBH73566	2	Susceptible	Broth microdilution (BrCAST)	Sanger (Conceição‐Neto et al. 2022)	Brazil	B	2016
CCBH73965	2	Susceptible	Broth microdilution (BrCAST)	Sanger (Conceição‐Neto et al. 2022)	Brazil	B	2016
12_BR_13	64	Resistant	Broth microdilution (CLSI)	SAMN04868732 (Pitt et al. 2018)	Brazil	B	2013
7610 I	1	Susceptible	Broth microdilution (BrCAST)	Sanger (This Work)	Brazil	B	2023
CBAS 537	0.5	Susceptible	Broth microdilution (BrCAST)	Sanger (This Work)	Brazil	B	2023
CBAS 540	2	Susceptible	Broth microdilution (BrCAST)	Sanger (This Work)	Brazil	B	2023
CBAS 541	1	Susceptible	Broth microdilution (BrCAST)	Sanger (This Work)	Brazil	B	2023
JC 22	1	Susceptible	Broth microdilution (BrCAST)	Sanger (This Work)	Brazil	B	2023
JC 23	2	Susceptible	Broth microdilution (BrCAST)	Sanger (This Work)	Brazil	B	2023
JC 31	1	Susceptible	Broth microdilution (BrCAST)	Sanger (This Work)	Brazil	B	2023
Kp37	2	Susceptible	Broth microdilution (BrCAST)	SAMEA4773886	Brazil	B	2016
kp71	2	Susceptible	Broth microdilution (BrCAST)	Sanger (Lourenço 2021)	Brazil	B	2016
10_GR_13	64	Resistant	Broth microdilution (CLSI)	SAMN04868730 (Pitt et al. 2018)	Greece	B	2013
13_GR_14	16	Resistant	Broth microdilution (CLSI)	SAMN04868733 (Pitt et al. 2018)	Greece	B	2014
18_GR_14	64	Resistant	Broth microdilution (CLSI)	SAMN04868738 (Pitt et al. 2018)	Greece	B	2014
19_GR_14	64	Resistant	Broth microdilution (CLSI)	SAMN04868739 (Pitt et al. 2018)	Greece	B	2014
23_GR_12	8	Resistant	Broth microdilution (CLSI)	SAMN04868743 (Pitt et al. 2018)	Greece	B	2012
4_GR_12	32	Resistant	Broth microdilution (CLSI)	SAMN04868724 (Pitt et al. 2018)	Greece	B	2012
7_GR_13	64	Resistant	Broth microdilution (CLSI)	SAMN04868727 (Pitt et al. 2018)	Greece	B	2013
8_GR_13	64	Resistant	Broth microdilution (CLSI)	SAMN04868728 (Pitt et al. 2018)	Greece	B	2013
9_GR_12	16	Resistant	Broth microdilution (CLSI)	SAMN04868729 (Pitt et al. 2018)	Greece	B	2012
ATH10	32	Resistant	Broth microdilution (CLSI)	SAMN12228971 (Zhu et al. 2019)	Greece	B	2016
ATH10	64	Resistant	Broth microdilution (CLSI)	SAMN12228971 (Zhu et al. 2019)	Greece	B	2016
ATH15	0,125	Susceptible	Broth microdilution (CLSI)	SAMN12228972 (Zhu et al. 2019)	Greece	E	2016
ATH16	128	Resistant	Broth microdilution (CLSI)	SAMN12228973 (Zhu et al. 2019)	Greece	B	2016
ATH17	0.25	Susceptible	Broth microdilution (CLSI)	SAMN12228974 (Zhu et al. 2019)	Greece	B	2016
ATH18	128	Resistant	Broth microdilution (CLSI)	SAMN12228975 (Zhu et al. 2019)	Greece	B	2016
ATH21	0.5	Susceptible	Broth microdilution (CLSI)	SAMN12228976 (Zhu et al. 2019)	Greece	B	2016
ATH22	32	Resistant	Broth microdilution (CLSI)	SAMN12228977 (Zhu et al. 2019)	Greece	B	2016
ATH23	0.5	Susceptible	Broth microdilution (CLSI)	SAMN12228978 (Zhu et al. 2019)	Greece	B	2016
ATH24	64	Resistant	Broth microdilution (CLSI)	SAMN12228979 (Zhu et al. 2019)	Greece	B	2016
ATH25	0.5	Susceptible	Broth microdilution (CLSI)	SAMN12228980 (Zhu et al. 2019)	Greece	B	2016
ATH26	64	Resistant	Broth microdilution (CLSI)	SAMN12228981 (Zhu et al. 2019)	Greece	B	2016
ATH30	32	Resistant	Broth microdilution (CLSI)	SAMN12228982 (Zhu et al. 2019)	Greece	B	2016
ATH8	128	Resistant	Broth microdilution (CLSI)	SAMN12228969 (Zhu et al. 2019)	Greece	B	2016
ATH9	0.5	Susceptible	Broth microdilution (CLSI)	SAMN12228970 (Zhu et al. 2019)	Greece	B	2016
AR_0125	4	Resistant	Broth microdilution (EUCAST)	SAMN04014966 (FDA/CDC)	Not mentioned	B	Not mentioned
kp19	16	Resistant	E‐test (BioMérieux)	SAMN06917612 (Rimoldi et al. 2017)	Italy	E	2012
kp34	16	Resistant	E‐test (BioMérieux)	SAMN06917627 (Rimoldi et al. 2017)	Italy	E	2013
kp36	0.5	Susceptible	E‐test (BioMérieux)	SAMN06917629 (Rimoldi et al. 2017)	Italy	E	2013
kp37	16	Resistant	E‐test (BioMérieux)	SAMN06917630 (Rimoldi et al. 2017)	Italy	E	2013
kp38	0.5	Susceptible	E‐test (BioMérieux)	SAMN06917631 (Rimoldi et al. 2017)	Italy	E	2013
kp46	0.5	Susceptible	E‐test (BioMérieux)	SAMN06917639 (Rimoldi et al. 2017)	Italy	E	2013
kp49	0.5	Susceptible	E‐test (BioMérieux)	SAMN06917642 (Rimoldi et al. 2017)	Italy	E	2013
AR_0363	0.5	Susceptible	Not mentioned	SAMN07291506 (FDA/CDC)	Not mentioned	B	Not mentioned
AR_0454	8	Resistant	Broth microdilution (CLSI)	SAMN07291547 (FDA/CDC)	Not mentioned	B	Not mentioned
Kp1001	0.25	Susceptible	Broth microdilution (EUCAST)	SAMEA110371303 (Elias et al. 2022)	Portugal	E	2004
Kp1003	0.25	Susceptible	Broth microdilution (EUCAST)	SAMEA110371304 (Elias et al. 2022)	Portugal	E	2004
Kp1019	0.25	Susceptible	Broth microdilution (EUCAST)	SAMEA110371306 (Elias et al. 2022)	Portugal	E	2004
Kp1031	0.25	Susceptible	Broth microdilution (EUCAST)	SAMEA110371322 (Elias et al. 2022)	Portugal	E	2005
Kp1032	0.25	Susceptible	Broth microdilution (EUCAST)	SAMEA110371323 (Elias et al. 2022)	Portugal	E	2005
Kp1036	0.25	Susceptible	Broth microdilution (EUCAST)	SAMEA110371324 (Elias et al. 2022)	Portugal	E	2005
Kp1122	0.25	Susceptible	Broth microdilution (EUCAST)	SAMEA110371327 (Elias et al. 2022)	Portugal	E	2005
Kp1144	0.25	Susceptible	Broth microdilution (EUCAST)	SAMEA110371346 (Elias et al. 2022)	Portugal	E	2006
Kp1209	0.25	Susceptible	Broth microdilution (EUCAST)	SAMEA110371347 (Elias et al. 2022)	Portugal	E	2006
Kp1363	0.25	Susceptible	Broth microdilution (EUCAST)	SAMEA6829111 (Elias et al. 2022)	Portugal	E	2005
Kp1495	0.25	Susceptible	Broth microdilution (EUCAST)	SAMEA110371363 (Elias et al. 2022)	Portugal	E	2007
Kp1495	0.25	Susceptible	Broth microdilution (EUCAST)	SAMEA110371363 (Elias et al. 2022)	Portugal	E	2007
Kp1507	1	Susceptible	Broth microdilution (EUCAST)	SAMEA110371364 (Elias et al. 2022)	Portugal	E	2007
Kp1528	0.25	Susceptible	Broth microdilution (EUCAST)	SAMEA110371385 (Elias et al. 2022)	Portugal	E	2008
Kp1675	0.25	Susceptible	Broth microdilution (EUCAST)	SAMEA6829114 (Elias et al. 2022)	Portugal	E	2008
Kp1924	2	Susceptible	Broth microdilution (EUCAST)	SAMEA110371699 (Elias et al. 2022)	Portugal	E	2018
Kp1938	1	Susceptible	Broth microdilution (Eucast)	SAMEA110371700 (Elias et al. 2022)	Portugal	E	2018
Kp1990	1	Susceptible	Broth microdilution (EUCAST)	SAMEA110371386 (Elias et al. 2022)	Portugal	E	2008
Kp2162	0.25	Susceptible	Broth microdilution (EUCAST)	SAMEA110371387 (Elias et al. 2022)	Portugal	E	2008
Kp2200	1	Susceptible	Broth microdilution (EUCAST)	SAMEA110371389 (Elias et al. 2022)	Portugal	E	2008
Kp2209	0.25	Susceptible	Broth microdilution (EUCASTst)	SAMEA6829115 (Elias et al. 2022)	Portugal	E	2008
Kp2224	1	Susceptible	Broth microdilution (EUCAST)	SAMEA110371702 (Elias et al. 2022)	Portugal	E	2008
Kp2287	1	Susceptible	Broth microdilution (EUCAST)	SAMEA110371702 (Elias et al. 2022)	Portugal	E	2018
Kp2334	1	Susceptible	Broth microdilution (EUCAST)	SAMEA110371391 (Elias et al. 2022)	Portugal	E	2008
Kp2447	1	Susceptible	Broth microdilution (Eucast)	SAMEA110371392 (Elias et al. 2022)	Portugal	E	2008
Kp2454	1	Susceptible	Broth microdilution (EUCAST)	SAMEA110371393 (Elias et al. 2022)	Portugal	E	2008
Kp2463	1	Susceptible	Broth microdilution (EUCAST)	SAMEA110371427 (Elias et al. 2022)	Portugal	E	2009
Kp2476	1	Susceptible	Broth microdilution (EUCAST)	SAMEA110371428 (Elias et al. 2022)	Portugal	E	2009
Kp2497	1	Susceptible	Broth microdilution (EUCAST)	SAMEA110371429 (Elias et al. 2022)	Portugal	E	2009
Kp2564	1	Susceptible	Broth microdilution (EUCAST)	SAMEA6829116 (Elias et al. 2022)	Portugal	E	2009
Kp2568	1	Susceptible	Broth microdilution (EUCAST)	SAMEA110371430 (Elias et al. 2022)	Portugal	E	2009
Kp2587	1	Susceptible	Broth microdilution (EUCAST)	SAMEA110371431 (Elias et al. 2022)	Portugal	E	2009
Kp2605	1	Susceptible	Broth microdilution (EUCAST)	SAMEA110371432 (Elias et al. 2022)	Portugal	E	2009
Kp2606	1	Susceptible	Broth microdilution (EUCAST)	SAMEA110371433 (Elias et al. 2022)	Portugal	E	2009
Kp2645	1	Susceptible	Broth microdilution (EUCAST)	SAMEA110371434 (Elias et al. 2022)	Portugal	E	2009
Kp2786	1	Susceptible	Broth microdilution (EUCAST)	SAMEA110371438 (Elias et al. 2022)	Portugal	E	2009
Kp2864	1	Susceptible	Broth microdilution (EUCAST)	SAMEA110371439 (Elias et al. 2022)	Portugal	E	2009
Kp2895	1	Susceptible	Broth microdilution (EUCAST)	SAMEA110371440 (Elias et al. 2022)	Portugal	E	2009
Kp2948	1	Susceptible	Broth microdilution (EUCAST)	SAMEA110371461 (Elias et al. 2022)	Portugal	E	2010
Kp2958	0.25	Susceptible	Broth microdilution (EUCAST)	SAMEA6829117 (Elias et al. 2022)	Portugal	E	2010
Kp3000	1	Susceptible	Broth microdilution (EUCAST)	SAMEA110371462 (Elias et al. 2022)	Portugal	E	2010
Kp3046	1	Susceptible	Broth microdilution (EUCAST)	SAMEA110371463 (Elias et al. 2022)	Portugal	E	2010
Kp3185	1	Susceptible	Broth microdilution (EUCAST)	SAMEA110371464 (Elias et al. 2022)	Portugal	E	2010
Kp3270	0.25	Susceptible	Broth microdilution (EUCAST)	SAMEA110371480 (Elias et al. 2022)	Portugal	E	2011
Kp3323	1	Susceptible	Broth microdilution (EUCAST)	SAMEA110371482 (Elias et al. 2022)	Portugal	E	2011
Kp3509	1	Susceptible	Broth microdilution (EUCAST)	SAMEA110371484 (Elias et al. 2022)	Portugal	E	2011
Kp3660	1	Susceptible	Broth microdilution (EUCAST)	SAMEA110371486 (Elias et al. 2022)	Portugal	E	2011
Kp3725	0.25	Susceptible	Broth microdilution (EUCAST)	SAMEA110371488 (Elias et al. 2022)	Portugal	E	2011
Kp3851	2	Susceptible	Broth microdilution (EUCAST)	SAMEA110371510 (Elias et al. 2022)	Portugal	E	2012
Kp3860	0.25	Susceptible	Broth microdilution (EUCAST)	SAMEA6829118 (Elias et al. 2022)	Portugal	E	2013
Kp4164	16	Resistant	Broth microdilution (EUCAST)	SAMEA110371233 (Elias et al. 2022)	Portugal	E	1980
Kp4194	0.25	Susceptible	Broth microdilution (EUCAST)	SAMEA110371261 (Elias et al. 2022)	Portugal	E	1982
Kp4195	1	Susceptible	Broth microdilution (EUCAST)	SAMEA110371262 (Elias et al. 2022)	Portugal	E	1982
Kp4197	0.25	Susceptible	Broth microdilution (EUCAST)	SAMEA110371263 (Elias et al. 2022)	Portugal	E	1982
Kp4246	0.5	Susceptible	Broth microdilution (EUCAST)	SAMEA110371236 (Elias et al. 2022)	Portugal	E	1980
Kp4256	0.25	Susceptible	Broth microdilution (EUCAST)	SAMEA110371240 (Elias et al. 2022)	Portugal	E	1980
Kp4265	0.5	Susceptible	Broth microdilution (EUCAST)	SAMEA110371247 (Elias et al. 2022)	Portugal	E	1981
Kp4279	0.25	Susceptible	Broth microdilution (EUCAST)	SAMEA110371264 (Elias et al. 2022)	Portugal	E	1982
Kp4287	0.25	Susceptible	Broth microdilution (EUCAST)	SAMEA110371265 (Elias et al. 2022)	Portugal	E	1991
Kp4292	0.25	Susceptible	Broth microdilution (EUCAST)	SAMEA110371266 (Elias et al. 2022)	Portugal	E	1995
Kp4333	0.25	Susceptible	Broth microdilution (EUCAST)	SAMEA110371269 (Elias et al. 2022)	Portugal	E	1995
Kp4367	0.25	Susceptible	Broth microdilution (EUCAST)	SAMEA110371270 (Elias et al. 2022)	Portugal	E	1995
Kp4387	0.25	Susceptible	Broth microdilution (EUCAST)	SAMEA110371272 (Elias et al. 2022)	Portugal	E	1995
Kp4408	0.25	Susceptible	Broth microdilution (EUCAST)	SAMEA110371274 (Elias et al. 2022)	Portugal	E	1996
Kp4855	1	Susceptible	Broth microdilution (EUCAST)	SAMEA110371528 (Elias et al. 2022)	Portugal	E	2013
Kp4856	1	Susceptible	Broth microdilution (EUCAST)	SAMEA110371537 (Elias et al. 2022)	Portugal	E	2014
Kp4857	1	Susceptible	Broth microdilution (EUCAST)	SAMEA110371538 (Elias et al. 2022)	Portugal	E	2014
Kp4860	2	Susceptible	Broth microdilution (EUCAST)	SAMEA110371541 (Elias et al. 2022)	Portugal	E	2014
Kp4861	2	Susceptible	Broth microdilution (EUCAST)	SAMEA110371542 (Elias et al. 2022)	Portugal	E	2014
Kp4862	1	Susceptible	Broth microdilution (EUCAST)	SAMEA110371543 (Elias et al. 2022)	Portugal	E	2014
Kp4864	1	Susceptible	Broth microdilution (EUCAST)	SAMEA110371561 (Elias et al. 2022)	Portugal	E	2015
Kp4865	1	Susceptible	Broth microdilution (EUCAST)	SAMEA110371562 (Elias et al. 2022)	Portugal	E	2015
Kp4869	1	Susceptible	Broth microdilution (EUCAST)	SAMEA110371490 (Elias et al. 2022)	Portugal	E	2011
Kp4871	1	Susceptible	Broth microdilution (EUCAST)	SAMEA110371566 (Elias et al. 2022)	Portugal	E	2015
Kp4878	1	Susceptible	Broth microdilution (EUCAST)	SAMEA110371573 (Elias et al. 2022)	Portugal	E	2015
Kp4882	2	Susceptible	Broth microdilution (EUCAST)	SAMEA110371582 (Elias et al. 2022)	Portugal	E	2016
Kp4886	1	Susceptible	Broth microdilution (EUCAST)	SAMEA110371585 (Elias et al. 2022)	Portugal	E	2016
Kp4887	1	Susceptible	Broth microdilution (EUCAST)	SAMEA110371586 (Elias et al. 2022)	Portugal	E	2016
Kp4889	16	Resistant	Broth microdilution (EUCAST)	SAMD00060934 (Elias et al. 2022)	Portugal	E	2016
Kp5505	16	Resistant	Broth microdilution (EUCAST)	SAMD00060934 (Elias et al. 2022)	Portugal	E	2016
Kp5506	16	Resistant	Broth microdilution (EUCAST)	SAMEA110371705 (Elias et al. 2022)	Portugal	E	2019
Kp5508	16	Resistant	Broth microdilution (EUCAST)	SAMEA110371706 (Elias et al. 2022)	Portugal	E	2019
Kp5509	16	Resistant	Broth microdilution (EUCAST)	SAMEA110371707 (Elias et al. 2022)	Portugal	E	2019
Kp5510	16	Resistant	Broth microdilution (EUCAST)	SAMEA110371703 (Elias et al. 2022)	Portugal	E	2018
Kp5511	16	Resistant	Broth microdilution (EUCAST)	SAMEA110371708 (Elias et al. 2022)	Portugal	E	2019
Kp5513	16	Resistant	Broth microdilution (EUCAST)	SAMEA110371709 (Elias et al. 2022)	Portugal	E	2019
Kp5514	16	Resistant	Broth microdilution (EUCASTcast)	SAMEA110371710 (Elias et al. 2022)	Portugal	E	2019
Kp5516	16	Resistant	Broth microdilution (EUCASTast)	SAMEA110371711 (Elias et al. 2022)	Portugal	E	2019
Kp5520	16	Resistant	Broth microdilution (EUCAST)	SAMEA110371713 (Elias et al. 2022)	Portugal	E	2019
Kp684	0.25	Susceptible	Broth microdilution (EUCAST)	SAMEA110371275 (Elias et al. 2022)	Portugal	E	1999
Kp689	0.25	Susceptible	Broth microdilution (EUCAST)	SAMEA110371276 (Elias et al. 2022)	Portugal	E	1999
Kp748	0.25	Susceptible	Broth microdilution (EUCAST)	SAMEA110371282 (Elias et al. 2022)	Portugal	E	2001
Kp776	0.25	Susceptible	Broth microdilution (Eucast)	SAMEA110371283 (Elias et al. 2022)	Portugal	E	2001
Kp804	0.25	Susceptible	Broth microdilution (Eucast)	SAMEA110371285 (Elias et al. 2022)	Portugal	E	2002
Kp828	0.25	Susceptible	Broth microdilution (EUCAST)	SAMEA110371287 (Elias et al. 2022)	Portugal	E	2002
Kp829	0.25	Susceptible	Broth microdilution (EUCAST)	SAMEA110371293 (Elias et al. 2022)	Portugal	E	2003
Kp840	1	Susceptible	Broth microdilution (EUCAST)	SAMEA110371294 (Elias et al. 2022)	Portugal	E	2003
Kp850	0.25	Susceptible	Broth microdilution (EUCAST)	SAMEA110371295 (Elias et al. 2022)	Portugal	E	2003
Kp874	0.25	Susceptible	Broth microdilution (EUCAST)	SAMEA110371296 (Elias et al. 2022)	Portugal	E	2003
Kp875	0.25	Susceptible	Broth microdilution (EUCAST)	SAMEA110371297 (Elias et al. 2022)	Portugal	E	2003
Kp888	0.25	Susceptible	Broth microdilution (EUCAST)	SAMEA110371298 (Elias et al. 2022)	Portugal	E	2003
Kp898	0.25	Susceptible	Broth microdilution (EUCAST)	SAMEA110371308 (Elias et al. 2022)	Portugal	E	2004
Kp910	0.25	Susceptible	Broth microdilution (EUCAST)	SAMEA110371309 (Elias et al. 2022)	Portugal	E	2004
Kp918	1	Susceptible	Broth microdilution (EUCAST)	SAMEA110371311 (Elias et al. 2022)	Portugal	E	2004
Kp919	1	Susceptible	Broth microdilution (EUCAST)	SAMEA110371312 (Elias et al. 2022)	Portugal	E	2004
Kp972	0.25	Susceptible	Broth microdilution (EUCAST)	SAMEA110371313 (Elias et al. 2022)	Portugal	E	2004
Kp986	0.25	Susceptible	Broth microdilution (Eucast)	SAMEA110371314 (Elias et al. 2022)	Portugal	E	2004
Kp997	0.25	Susceptible	Broth microdilution (EUCAST)	SAMEA110371315 (Elias et al. 2022)	Portugal	E	2004

### 2.2 Cultivation, DNA Purification, PCR Amplification and Sanger Sequencing

Twenty‐one isolates were grown on MacConkey agar plates. Pure colonies were subcultured in brain heart infusion (BHI) broth and incubated at 37°C under agitation (180 rpm) for 16 h. Cryopreservation was performed using a 15% glycerol stock (270 μL 99% glycerol + 1530 μL overnight culture) stored at −80°C. For species reconfirmation, isolates were streaked onto nutrient agar, and pure colonies were transferred to a target plate for matrix‐assisted laser desorption/ionisation time‐of‐flight (MALDI‐TOF) mass spectrometry analysis at the National Institute of Infectious Diseases (INI) platform, using the MALDI TOF‐MS Biotyper system (Bruker Daltonics, v4.1.3). Genomic DNA was purified using the QIAamp DNA Mini Kit (Qiagen, v2023) and eluted in 30 μL of nuclease‐free water. Primers targeting *mgrB*, *phoP*, *phoQ*, *pmrA* and *pmrB* and other molecular biology protocols are detailed in Data S1. Amplification was performed using GoTaq Green Master Mix 2× (Promega, v2.0) under optimised thermocycling conditions (Data S1). PCR products were purified with the QIAquick PCR Purification Kit (Qiagen, v2023) and eluted in 30 μL. DNA concentration and purity were assessed using a NanoDrop One spectrophotometer (Thermo Fisher Scientific, v1.12) with A260/A280 and A260/A230 ratios. Sanger sequencing of amplicons was conducted at the FIOCRUZ Genomics Platform using a 3730xl DNA Analyser (Applied Biosystems, v5.0) with BigDye Terminator v3.1 chemistry (Thermo Fisher Scientific, v3.1).

### 2.3 Acquisition of *mgrB* , *phoP* , *phoQ* , *pmrA* , *pmrB* and *rpoB* Sequences

Whole Genome Shotgun (WGS) sequence data of 145 isolates were retrieved from the National Center for Biotechnology Information (NCBI) via BioProjects PRJNA307517 (Pitt et al. 2018), PRJEB38289 (Elias et al. 2022), PRJNA385863 (Rimoldi et al. 2017) and PRJNA316321 (FDA/CDC Antimicrobial Resistant Isolate Bank) (NCBI Resource Coordinators 2018). For isolates with RefSeq assemblies, coding sequences (*mgrB*, *phoP*, *phoQ*, *pmrA*, *pmrB, rpoB*) were directly extracted from annotated GenBank files. For isolates with raw Sequence Read Archive (SRA) data, FASTQ files were generated using fastq‐dump (SRA Toolkit v3.0.0) (Leinonen et al. 2011). Low‐quality reads and adapters were trimmed using fastp (v0.23.4) (Chen et al. 2018). For FASTq reads from the SRA database, gene coverage was calculated based on the single‐copy gene rpoB as a reference for 98 genomes using the bowtie2 mapper and samtools. For 40 assemblies available in Refseq and Genbank, the genomic coverage values reported by the submitters were used directly. The estimated genomic coverage for SRA and Assembly (Genbank/Refseq) data was similar, 91.2× and 102.2×, respectively. The standard deviation of SRA data from Illumina sequences was lower (about 22.6) when compared to that of assemblies (of 82.0) (Table S1). The reference genome *K. pneumoniae* MGH78578 (GCF_000016305.1) was downloaded from NCBI GenBank. Functional annotation of raw reads was performed using Prokka (v1.14.6) (Seemann 2014), generating GFF annotation files. Filtered reads were aligned to the reference genome using BWA‐MEM (v0.7.17) (Li 2013) and resulting BAM files were sorted and indexed with Samtools (v1.17) (Danecek et al. 2021). Coding regions were extracted using BEDTools getfasta (v2.31.0) (Quinlan and Hall 2010), enabling gene‐specific sequence recovery from metagenomic alignments. For the twenty‐one isolates processed in LabSUR, *mgrB*, *phoP*, *phoQ*, *pmrA* and *pmrB* sequence data was obtained by Sanger sequencing. Forward and reverse reads (including internal primers for *phoQ*) were aligned to the *K. pneumoniae* MGH78578 reference genes using Geneious Prime (v2023.1.1) (Kearse et al. 2012), with consensus sequences generated via built‐in error correction algorithms. For neutral marker analysis, the *rpoB* fragment used in Multi Locus Sequence Type (MLST) analysis of *K. pneumoniae* isolates was chosen (position 1657 to 2157; Diancourt et al. 2005) (Table S2). For thirty‐six isolates, *rpoB* allele information was inferred based on the sequence type (ST) reported in the original reference (Conceição Neto et al. 2022). The corresponding *rpoB* alleles were subsequently retrieved from the *Klebsiella pneumoniae* MLST database hosted at the Institut Pasteur.

### 2.4 Detection of Nonsynonymous Substitutions

Multi‐FASTA files were generated for *mgrB*, *phoP*, *phoQ*, *pmrA* and *pmrB*, encompassing all 214 isolates. Multiple sequence alignment (MSA) was performed using MEGA11 (v11.0.13) (Kumar et al. 2021) with the ClustalW algorithm under default parameters. Alignments were conducted for both nucleotide sequences and their translated amino acid counterparts. The amino acid MSA was specifically analysed to identify nonsynonymous substitutions relative to the wild‐type sequences of the *K. pneumoniae* MGH78578 reference genome (GCF_000016305.1) (NCBI Resource Coordinators 2018). Positions exhibiting amino acid deviations from the reference were annotated as putative nonsynonymous mutations, with manual validation to exclude alignment artefacts.

### 2.5 Allelic Screening, Mapping and Haplotype Network Construction

Nonsynonymous substitutions were consolidated into a unified table integrating data from all five genes (*mgrB*, *phoP*, *phoQ*, *pmrA*, *pmrB*) for each isolate, with haplotypes and their allele frequencies annotated. A custom Python script concatenated the multiple sequence alignments of these genes into a single contiguous DNA sequence. For isolates harbouring an insertion in *mgrB*, a guanine residue was manually added at position 124 to maintain reading frame consistency. The concatenated alignment was analysed in DnaSP v6.12.03 (Rozas et al. 2017) using the ‘Generate Haplotype Data’ function, which excluded invariant sites and generated a Roehl Data File (.rdf) for dow nstream analysis. The median‐joining algorithm (Bandelt et al. 1999) was implemented in Network v10.2 (Fluxus Technology Ltd. 2023) to construct the haplotype network, optimising branch connections based on mutational steps and genetic distances.

### 2.6 Subpopulation Definition and Neutrality Tests

The 214 isolates were stratified into seven subpopulations based on polymyxin susceptibility phenotypes and *mgrB* genetic profiles: (1) polymyxin‐susceptible (PM‐S), (2) polymyxin‐resistant (PM‐R), (3) isolates with MICs of 0.25–2.0 mg/L, (4) MICs of 4.0–16.0 mg/L, (5) MICs of 32.0–128.0 mg/L, (6) *mgrB* wild‐type and (7) *mgrB*‐altered (e.g., mutations, insertions, truncations, deletions). Neutrality tests were conducted in DnaSP v6.12.03 (Rozas et al. 2017) to assess evolutionary pressures acting on the five resistance genes and of a neutral marker (*rpoB* MLST fragment; Diancourt et al. 2005). Tajima's *D* (Tajima 1989) and Fu & Li's *D* (Fu and Li 1993) were calculated for each subpopulation using segregating sites (polymorphic positions). Negative values (*D* < 0) indicated potential positive selection or population expansion, while positive values (*D* > 0) suggested balancing selection or population subdivision.

## 3 Results

### 3.1 Isolates´ Distribution

The 214 isolates originated from four countries: Brazil (32.71%, *n* = 70), Greece (11.68%, *n* = 25), Italy (3.27%, *n* = 7) and Portugal (50.93%, *n* = 109) (NCBI Resource Coordinators 2018; BioProject accessions: PRJNA307517, PRJEB38289, PRJNA385863, PRJNA316321) (Table 1). Whole‐genome sequencing (WGS) had been performed on 145 isolates (67.76%), all sourced from European countries (Greece, Italy, Portugal), whereas sequences were obtained by Sanger sequencing in the case of the 69 Brazilian isolates (32.24%) (Table 1). The overrepresentation of isolates from Portugal (50.9%) and Brazil (32.7%) may skew the analysis and impact the generalisation of the results. To address this point, we looked into the ST distribution of the isolates in the dataset, as this is used to establish clonal relations and phylogeny of *K. pneumoniae*. We found that the five most frequent STs (ST15, 16.9%; ST258, 14.6%; ST11, 6.7%; ST147, 6.8%; ST437, 6.2%) accounted for 51.1% of the 178 isolates for which we could access *rpoB* fragment sequences. ST15, ST258, ST11 and ST147 are recognised as high‐risk clones with global distribution, often associated with multidrug resistance and hospital outbreaks in different continents (Wyres et al. 2020; Lam et al. 2021 in this reference see Figures S5 and S6; Arcari and Carattoli 2022; Wu et al. 2023; Szijártó et al. 2016; Rodrigues et al. 2022). ST437 is a single‐locus variant of the pandemic clone ST258, and though its distribution is global (Sahoo et al. 2023), its frequency increase has been documented in Europe (Budia‐Silva et al. 2024). In summary, the sampling of isolates from four countries broadly matches the profile of STs with global dissemination, though the representation of some STs may be skewed (as is the case for ST437).

### 3.2 Susceptibility Profile

The study population comprised 123 alleles (57.48%) associated with polymyxin susceptibility, and 91 alleles (42.52%) linked to resistance (CLSI 2023) (Table S3). Within the susceptible cohort (*n* = 123), 77 alleles (62.6%) exhibited no nonsynonymous substitutions, while the remaining 46 alleles (37.4%) harboured ≤ 1 mutation. In contrast, 90 resistant alleles (98.9%) carried ≥ 1 genetic alteration, with single (*n* = 65, 71.4%) and double (*n* = 28, 30.8%) mutations predominating (Moffatt et al. 2010). Among *mgrB* wild‐type isolates (*n* = 145), 84.83% (*n* = 123) were susceptible, whereas 15.17% (*n* = 22) displayed resistance despite intact *mgrB*. All *mgrB*‐altered isolates (*n* = 69) were resistant, with 92.75% (*n* = 64) exhibiting high‐level resistance (MIC ≥ 16.0 mg/L). MIC‐based stratification revealed three subpopulations: susceptible (0.25–2.0 mg/L, *n* = 122), intermediate resistance (4.0–16.0 mg/L, *n* = 35) and high resistance (32.0–128.0 mg/L, *n* = 56) (Table S3).

### 3.3 Genetic Diversity of Haplotypes

**FIGURE 1:**
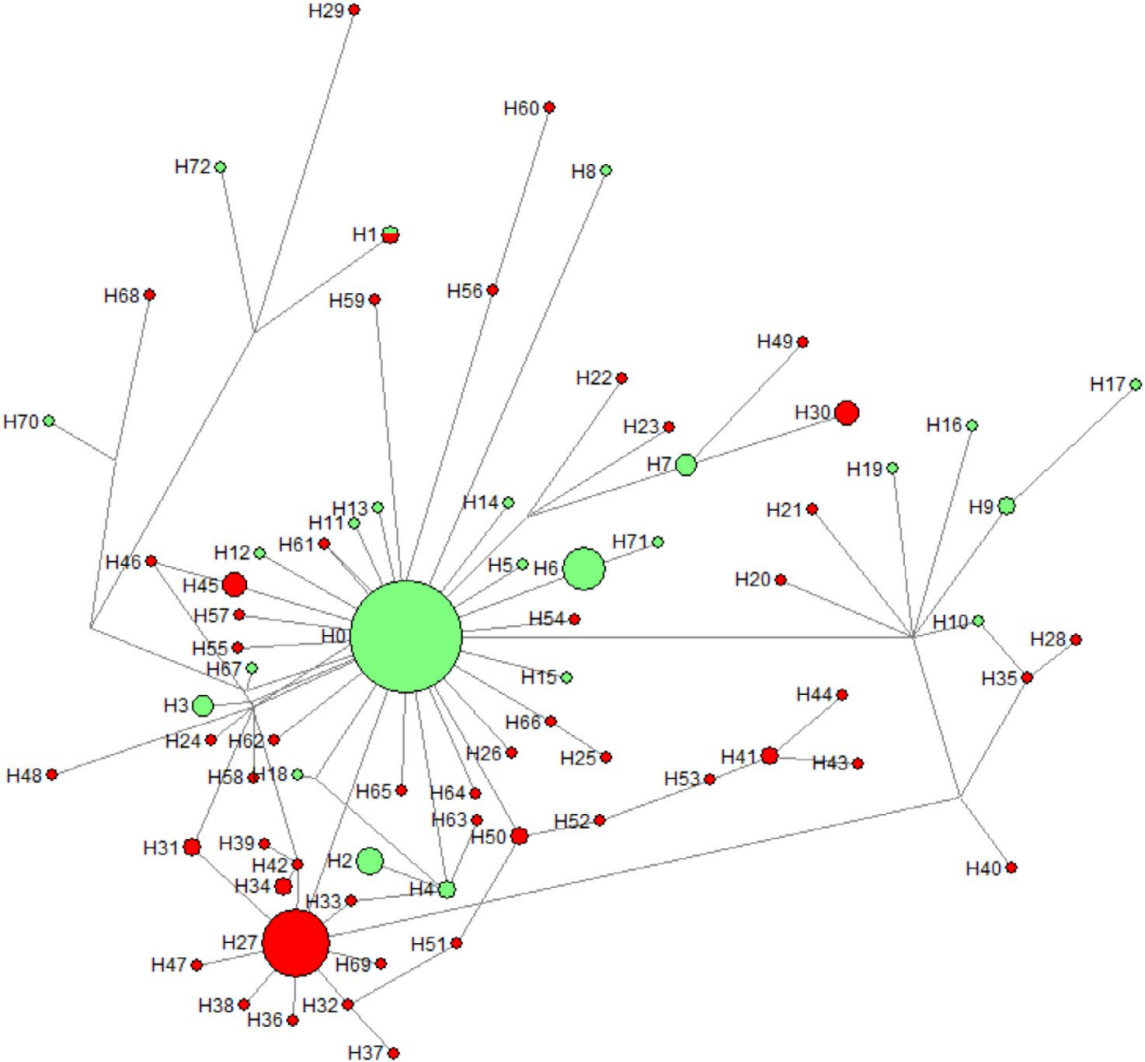
Haplotype network of all alleles. Green colours related to susceptible phenotype and red ones resistant. Size of circumference is proportional to *N*.

**FIGURE 2:**
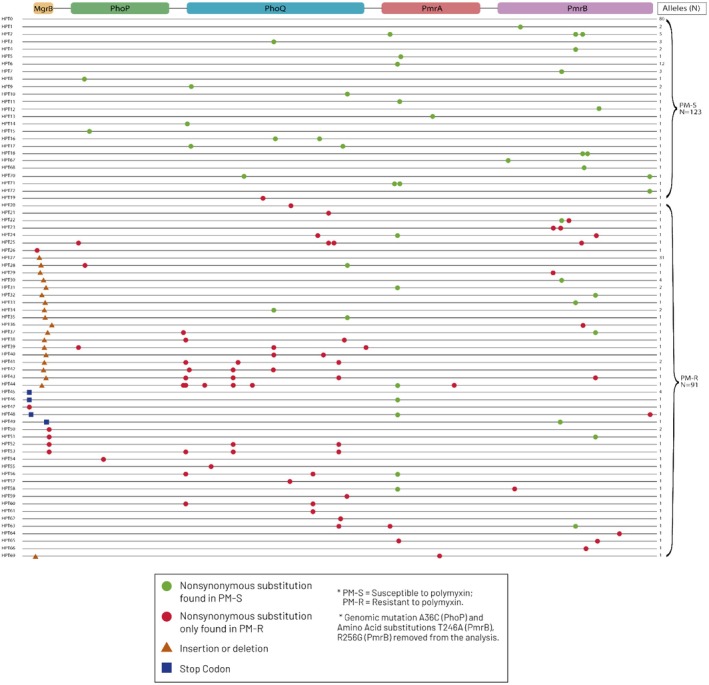
Diagram for visualisation of changes in MgrB, PmrAB and PhoPQ according to haplotype. The number of isolates in which each haplotype was identified is shown on the right.

A total of 73 haplotypes were identified among the 214 analysed, with 23 associated with susceptibility and 49 linked to resistance (Figure 1; Table 2). Haplotypes 1 and 2 harboured alleles exhibiting both phenotypes. Resistance‐associated haplotypes displayed 49 unique variations, while susceptible haplotypes contained 25. Eighty alleles (37.4%) were identical to the reference genome (haplotype 0), indicating conserved sequences (Figure 1; Table 2; Table S3). Haplotype 27, the second most frequent (31 alleles), was exclusively resistant and characterised solely by *mgrB* insertions. Notably, 59 haplotypes (79.7%) were singletons, reflecting rare alleles (27.57% of the total) (Figure 2).

**TABLE 2:** Genetic diversity of haplotypes.

Haplotype	MgrB^a^	PhoP	PhoQ	PmrA	PmrB^a^	Allele copies	Phenotype
0						80	S
1					T140P	2	S/R
2				A41Y	T240M, L213M	5	S/R
3			N253T			3	S
4					L213M	2	S
5				M66I		1	S
6				E57G		12	S
7					M175V	3	S
8		A110S				1	S
9			S56R			2	S
10			V446G			1	S
11				G53S		1	S
12					A282S	1	S
13				I127V		1	S
14			L28F			1	S
15		D135N				1	S
16			N255I, S350Y			1	S
17			S56R, I422S			1	S
18					T240M, V257A	1	S
19			L239P			1	S
20			T281M			1	R
21			G385C			1	R
22					M175V, G207D	1	R
23					T157P, M175V	1	R
24			H339D	E57G		1	R
25		S72L	S409R. H410Y		V280L	1	R
26	W20R					1	R
27	Insertion					31	R
28	Insertion	A95S	V446G			1	R
29	Insertion				P158R	1	R
30	Insertion				M175V	4	R
31	Insertion			E57G		2	R
32	Insertion				A282R	1	R
33	Insertion				L213M	1	R
34	Insertion		N253P			2	R
35	Insertion		V446G			1	R
36	Insertion				L222A	1	R
37	Insertion		R16C		A282R	1	R
38	Insertion		V27H, F398K			1	R
39	Insertion	P74L	N253T			1	R
40	Insertion		N253T, T439P			1	R
41	Insertion		V27H, P103W, C395A			2	R
42	Insertion		G39S, A225T, N253T			1	R
43	Insertion		V27H, P103W, C395A		A282R	1	R
44	Insertion		R16A, V27H, D73I, P103W, S188T	R160S, E57G		1	R
45	K3Stop					4	R
46	K3Stop			E57G		1	R
47	K3T					1	R
48	L8Stop			E57G	G345R	1	R
49	Q30Stop				M175V	1	R
50	S36R					2	R
51	S36R				A282R	1	R
52	S36R		P103W, C395A			1	R
53	S36R		V27H, P103W, C395A			1	R
54		G121A				1	R
55			I88N			1	R
56			V27H, Y265C	E57G		1	R
57			T246C			1	R
58				E57G	H61Q	1	R
59			Q405A			1	R
60			V27H, Y265T			1	R
61			Y265C			1	R
62			E397G			1	R
63			C395V	A41T	L213M	1	R
64					M285L	1	R
65				L63H		1	R
66					V280L	1	R
67					E7Q	1	S
68					T240M	1	S
69	I			L132M		1	R
70			D150G		P344L	1	S
71				E35A, M66I		1	S
72					P346Q	1	S

^a^ Nucleotidic mutation A36C in *mgrB* and substitutions T246A and R256G in PmrB were removed to avoid bias in neutraliy tests.

### 3.4 Neutrality Tests Across Subpopulations

**FIGURE 3:**
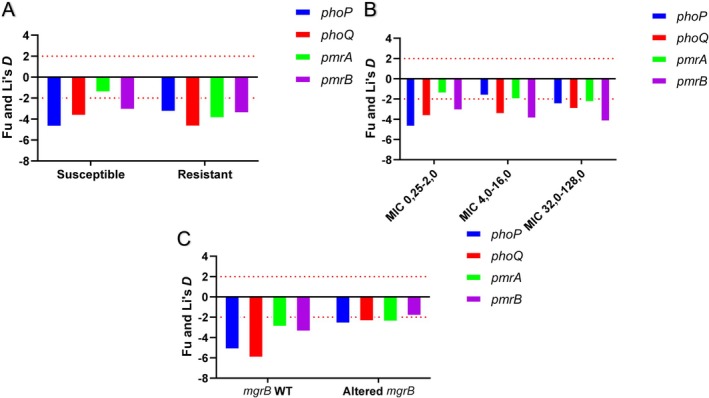
(A) Fu and Li's *D* value for polymyxin susceptible (PM‐S) and resistant (PM‐R) populations. (B) Fu and Li's *D* value for MIC intervals populations. (C) Fu and Li's *D* value for *mgrB* wild type and altered *mgrB* populations. Bar colours are related to the gene value.

Analysis of Tajima's *D*, Fu & Li's *D* and haplotype diversity (HD) revealed distinct evolutionary patterns among subpopulations (Table 3). In the polymyxin‐susceptible (PM‐S) group (Figure 3), *phoP*, *phoQ* and *pmrB* exhibited significantly negative Tajima's *D* values (range: −1.98621 to −2.48068; *p* < 0.05 to *p* < 0.01), with even lower Fu & Li's *D* values (−3.17819 to −4.78393) (Tajima 1989; Fu and Li 1993). No substitutions were detected in *mgrB* within PM‐S, precluding neutrality calculations for this gene. The *D* values for *pmrA* did not show significant deviations from neutrality, and HD remained low (0–0.28). The polymyxin‐resistant (PM‐R) subpopulation (Figure 3) displayed similar trends, except for *phoQ*, which showed a more pronounced Fu & Li's *D* (−4.77326 vs. −3.75183 in PM‐S). Notably, PM‐R exhibited higher haplotype diversity (HD = 0.406–0.844). *mgrB* *D* values did not deviate significantly from neutrality. Subpopulations stratified by MIC intervals (Figure 3) mirrored PM‐S and PM‐R patterns. Low‐MIC isolates (0.25–2.0 mg/L) mirrored PM‐S in *D* and HD values. However, intermediate‐ (4.0–16.0 mg/L) and high‐MIC (32.0–128.0 mg/L) groups revealed divergent trends: *phoQ* Tajima's *D* increased with MIC (*p* < 0.05), while *pmrB* values decreased (*p* < 0.01). HD peaked in intermediate‐MIC isolates before slightly declining in high‐MIC groups. Comparative analysis of *mgrB* wild type versus altered subpopulations (Figure 3) highlighted stark contrasts. Wild‐type isolates showed markedly lower Fu & Li's *D* values (*phoP*: −5.22767 vs. −2.67576; *phoQ*: −6.03302 vs. −2.45506) alongside elevated HD (Table 3). Analysis of a neutral marker, the *rpo*B fragment used in MLST of *K. pneumoniae*, yielded Tajima's *D* value of −0.80752 and Fu and Li's *D* value of −0.14645 (Table 3). Albeit these values are negative, the P values are above 0.05, which is coherent with this sequence not deviating neutrality.

**TABLE 3:** *D* values and evolutionary analysis.

Locus	Subpopulation	Alleles (*N*)	Mutations (*N*)	Haplotypes (*N*)	HD	*π*	Tajima *D*	Fu and Li's *D*
*mgrB*	PM‐S	123	0	1	0	0	—	—
*phoP*			6	4	0.049	0.00015	−198621	−4.78393
*phoQ*			14	10	0.173	0.00037	−21106	−3.75183
*pmrA*			7	7	0.28	0.00266	−161927*	−1.51506*
*pmrB*			21	12	0.259	0.0028	−2.28478	−3.17757
*rpoB*		103	5	6	0.490	0.00116	−0.83934*	−1.09903*
*mgrB*	PM‐R	91	3	4	0.464	0.00387	−0.10052*	−0.56404*
*phoP*			3	4	0.065	0.0001	−0.10052*	−0.56404*
*phoQ*			38	22	0.49	0.00136	−1.62177*	−3.36191
*pmrA*			8	7	0.242	0.00047	−2.18456	−4.77326
*pmrB*			22	14	0.406	0.00086	−1.83389	−3.97162
*rpoB*		78	2	3	0.393	0.00082	−2.32146	−3.50909
*mgrB*	MIC 0.25–2.0	122	0	1	0	0	—	—
*phoP*			6	4	0.049	0.00015	−1.98621	−4.78393
*phoQ*			14	10	0.173	0.00037	−2.1106	−3.75183
*pmrA*			7	7	0.28	0.00056	−1.61927*	−1.51506*
*pmrB*			21	12	0.259	0.0007	−2.28478	−3.17757
*mgrB*	MIC 4.0–16.0	35	3	4	0.499	0.00401	−0.46554*	−1.55209*
*phoP*			1	2	0.057	0.00009	−1.13552*	−1.73221*
*phoQ*			16	8	0.365	0.00073	−2.38341	−3.54379
*pmrA*			5	5	0.393	0.00071	−1.25028*	−2.08249*
*pmrB*			21	12	0.745	0.00108	−2.05115	−3.62414
*mgrB*	MIC 32.0–128.0	56	2	3	0.447	0.00382	0.45975*	0.736*
*phoP*			2	3	0.071	0.00011	−1.45172*	−2.58453
*phoQ*			29	16	0.563	0.00173	−1.84334	−3.03588
*pmrA*			4	4	0.138	0.00031	−1.67941*	−2.36802*
*pmrB*			13	7	0.266	0.00055	−2.3188	−4.26335
*mgrB*	*mgrB* WT	145	0	1	0	0	—	—
*phoP*			7	5	0.057	0.00015	−2.05404	−5.22767
*phoQ*			29	21	0.29	0.00055	−2.44693	−6.03302
*pmrA*			9	9	0.316	0.00062	−1.64261*	−2.99296
*pmrB*			25	16	0.313	0.0008	−2.31222	−3.46716
*mgrB*	*mgrB* Altered	69	3	4	0.19	0.00243	−0.84579*	−0.49716*
*phoP*			2	3	0.058	0.00009	−1.42652*	−2.67576
*phoQ*			26	11	0.387	0.00133	−1.89522	−2.45506
*pmrA*			4	4	0.165	0.00033	−1.55338*	−2.48398
*pmrB*			15	8	0.36	0.00084	−2.0511	−1.91352*
*rpoB*	PM‐S and PM‐R	181	5	7	0,447	0.00101	−0.80752*	−0.14645*

Abbreviations: HD, haplotype diversity; *π*, nucleotide diversity.

* *D* values not significant (*p* > 0.05).

## 4 Discussion

### 4.1 Polymyxin Resistance Is Linked to Polygenic Adaptation

This study revealed high genetic diversity in *mgrB*, *phoP*, *phoQ*, *pmrA* and *pmrB* under varying polymyxin concentrations, attributed to the direct role of these five genes in mediating the addition of PEtN and L‐Ara4N to the lipid A moiety of LPS. Naturally negatively charged, the incorporation of these molecules alters LPS charge, hindering polymyxin binding to the cell wall (Jayol et al. 2014). *mgrB* plays the most prominent role in resistance (Poirel, Jayol, Bontron, et al. 2015), consistent with our findings, where all variations in this gene were identified in the PM‐R subpopulation. Following in relevance, *phoQ* and *pmrB* contribute significantly, while *phoP* and *pmrA* also influence resistance, albeit with lesser impact (Olaitan et al. 2014). The high diversity of mutations, insertions and deletions was quantified using Tajima's *D* and Fu & Li's statistics. Notably, all genes exhibited significantly negative values in both polymyxin‐resistant and susceptible populations. More negative *D* values indicate a higher proportion of segregating sites (Tajima) and rare alleles (Fu & Li), with many alleles showing substitutions at multiple *loci* (Ferretti et al. 2017). These findings suggest resistance‐related selective pressures across distinct *loci*, particularly as *mgrB*, *phoPQ* and *pmrAB* are genomically distant in *K. pneumoniae* . This process strongly parallels polygenic adaptation (Pritchard et al. 2010).

The concept of polygenic adaptation emerged over the 20th century. Ronald Fisher established the mathematical foundation for polygenic traits, proposing that continuous phenotypes like height are governed by multiple *loci* with small additive effects (Fisher 1918). In 1983, Russel Lande developed models integrating complex trait genetics with molecular evolution, demonstrating how selection acts on traits controlled by multiple *loci* and formalising additive genetic variance and covariance between loci shape species adaptation. The explicit term ‘polygenic adaptation’ was first introduced in Pritchard et al.'s 2010 review, which posited that this evolutionary process involves concurrent selection of genetic variants distributed across multiple genomic *loci*. Polygenic adaptation could also arise from new mutations at diverse *loci*, particularly if a newly favoured phenotype was previously strongly disadvantageous.

The latter scenario may explain the high frequency of substitutions and insertions observed in MgrB, PhoPQ and PmrAB. These proteins naturally modulate LPS charge to confer defence under low/high pH or metal ion scarcity. However, amino acid structural changes may lead to their hyperactivation—via inactivation of MgrB (a negative regulator of PhoQ) or enhanced phosphorylation sites in PhoQ and PmrB (Olaitan et al. 2014). Such alterations render the phenotype disadvantageous in environments lacking polymyxin pressure or with normal pH and Ca^2+^ levels (Janssen et al. 2020). The phenotypic preference observed in our study highlights a clear association between resistance and the abundance of variations in MgrB, PhoPQ and PmrAB, indicating polygenic adaptation in resistant populations. Other genes, such as those within the *arn* operon or *pmrCAB*, are also linked to polymyxin resistance. However, these were not analysed here, necessitating future investigation to confirm whether mutations in these loci synergistically alter resistance phenotypes alongside the studied genes.

### 4.2 The Studied Population Is Undergoing Recent Expansion

The results demonstrated very low Tajima's *D* values in polymyxin‐susceptible subpopulations. Two lines of evidence may explain these low values. First, there may be a bias due to the high frequency of alleles associated with haplotype 0 (*N* = 80). Haplotype 0 matches the reference genome used in this study and can be considered the ancestral haplotype of the entire population (Bandelt et al. 1999). Low *D* values are linked to a disproportion between haplotype frequencies and an excess of rare alleles in the subpopulation, with 17 out of 23 PM‐S haplotypes (73.91%) having *N* = 1. These data suggest a population expansion in PM‐S, as novel alleles are emerging from the ancestral haplotype, even at low frequencies.

Unlike Tajima's D, Fu & Li's statistics exhibit greater sensitivity to recent population expansion events. This is because Fu & Li's mathematical model weights segregating sites by singletons, which the authors proposed are linked to recent expansions, as such alleles have not yet had sufficient time to increase in frequency. The high number of singletons in PM‐S reflects a rapid population expansion of alleles carrying mutations in polymyxin resistance‐associated genes. Notably, polymyxins were officially reintroduced for treating hospital‐acquired infections around 2010, when they were recommended as a last‐resort option for infections caused by carbapenem‐resistant bacteria (Landman et al. 2008). Given that most strains analysed here date to the 2010s, the emergence of singletons may coincide with this period, reflecting a transitional spatiotemporal stage where these alleles have neither become fixed nor purged. A second supporting observation is that even minimal inhibitory concentrations (MICs) ≤ 2 μg/mL—associated with susceptibility—appear sufficient to promote adaptive emergence of new alleles, even if they are not directly linked to resistance. However, robust evidence for active selection in PM‐S is lacking, as the ancestral haplotype remains dominant and unrelated to polymyxin adaptation.

A similar pattern is observed in PM‐R, with low *D* values and the prevalent haplotype 27 (*N* = 31) followed by 41 rare alleles. Intriguingly, two primary expansion events underline resistant allele origins. The first stems from the ancestral haplotype, giving rise to multiple rare resistant haplotypes (e.g., haplotypes 45, 54 and 65). The second originates from haplotype 27 itself, which diverges from the ancestral lineage and spawns additional rare haplotypes (e.g., 32, 33, 47, 69) (Figure 1). Thus, two distinct expansion events occurred in the overall population: one in PM‐S arising from the ancestral haplotype and a second in PM‐R originating from haplotype 27. The latter event exclusively produced PM‐R alleles, highlighting divergent evolutionary trajectories under selective pressure.

### 4.3 Alterations in *mgrB* Drive Positive Selection via Selective Sweeps

The analysed subpopulations exhibit Fu & Li's *D* values below −3.0, which is indicative of extreme population expansion. Intriguingly, the *mgrB*‐altered subpopulation showed *D* values ranging from −1.91352 to −2.67576 (Fu & Li) and −1.42652 to −2.0511 (Tajima) for significant comparisons. While these values are less extreme, they remain notably elevated, suggesting potential positive selection acting on *mgrB*. Allelic frequency analysis revealed a decline in rare alleles with substitutions in *phoP*, *phoQ*, *pmrA* and *pmrB* when compared to haplotype 27 (*N* = 31/68), which dominates nearly half of the subpopulation. Haplotype 27 carries insertions such as ISKpn26, which inactivate *mgrB* as a negative regulator of histidine kinases (HKs), leading to LPS charge modification via hyperactivation (Jayol et al. 2014). This haplotype lacks mutations in *phoP*, *phoQ*, pmrA or *pmrB*, potentially explaining its higher *D* value. The high frequency of haplotype 27 implies it is sweeping the population through selective sweep dynamics, a hallmark of positive selection (Pritchard et al. 2010; Booker et al. 2017).

Selective sweeps occur when an allele confers a significant adaptive advantage, increasing its frequency across generations while reducing genetic diversity. These are categorised as ‘hard’ (complete or incomplete) or ‘soft’ sweeps. Hard sweeps involve a single advantageous mutation rising to fixation, dragging linked genomic regions along due to reduced recombination (classic model). Incomplete hard sweeps reflect ongoing fixation processes. Soft sweeps arise when preexisting neutral alleles become advantageous under new selective pressures or when multiple independent mutations confer similar benefits, preserving genetic diversity (Booker et al. 2017). Polygenic adaptation, as previously discussed, can also drive partial sweeps, where selection acts gradually across *loci*, maintaining diversity.

Our findings indicate that haplotype 27 is sweeping the PM‐R subpopulation via horizontal gene transfer (HGT)‐mediated insertion in *mgrB* rather than point mutation. HGT‐driven sweeps can propagate adaptive alleles faster than clonal reproduction (Wilson et al. 2016), potentially explaining haplotype 27's rapid dominance. Conversely, clonally derived haplotypes with mutations reflect partial sweeps linked to polygenic adaptation, sustaining genetic diversity.

The observed diversity may also stem from compensatory mutations unrelated to direct resistance mechanisms. These mutations mitigate fitness costs imposed by primary adaptations, such as LPS modifications, enabling resistant strains to remain competitive in non‐selective environments (Levin et al. 2000). Additionally, genetic hitchhiking—where neutral or deleterious mutations rise in frequency due to linkage with advantageous loci—may influence diversity in *mgrB*, *phoPQ* and *pmrAB*. This phenomenon, first described by Maynard Smith and Haigh (1974), is exemplified in *Mycobacterium tuberculosis* , where non‐resistance mutations fixed sequentially alongside adaptive alleles during antibiotic treatment (Eldholm et al. 2014).

Collectively, these findings suggest PM‐R is undergoing positive selection via *mgrB*‐targeted sweeps. Whether this sweep progresses toward fixation or remains incomplete hinges on polygenic adaptation or compensatory evolution, which could sustain genetic diversity through persistent low‐frequency alleles. Such dynamics underscore the complex interplay between selection, genetic linkage and adaptive trade‐offs in shaping resistance evolution.

### 4.4 Impact of Findings on Healthcare and Hospital Environments

Recent epidemiological data indicate that polymyxin resistance in *K. pneumoniae* has a global prevalence of approximately 11.64%, with regional variations: 10.17% in Asia, 16.16% in Europe and 18.67% in the Americas (Sameni et al. 2022). In Brazil, we previously reported that 29% of carbapenem‐resistant *K. pneumoniae* (CRKP) isolates from diverse regions exhibited resistance to polymyxin B (Conceição‐Neto et al. 2022). As CRKP remains a top priority for surveillance and research (WHO 2024), polymyxin use is likely to escalate in coming decades unless alternative therapies are integrated into clinical protocols.

Our study highlights a rapid population expansion of resistant strains following the reintroduction of polymyxins in hospital settings. This expansion originates from both the ancestral haplotype (haplotype 0) and the *mgrB*‐altered haplotype 27. In the case of haplotype 27, selective sweeps are driving its overrepresentation among resistant populations. If these sweeps proceed to completion, this haplotype is expected to dominate hospital environments, with rare alleles failing to establish.

However, mechanisms such as compensatory evolution may impede complete sweeps. Outside high‐polymyxin environments, the fitness cost of a positively charged LPS reduces strain competitiveness (Janssen et al. 2020). Rare alleles derived from haplotype 27 (e.g., haplotypes 28–44) encode amino acid substitutions in *phoP*, *phoQ*, *pmrA* and *pmrB*, potentially attenuating histidine kinase (HK) activity in PhoQ and PmrB despite their overexpression. These mutations may downregulate *arn* operon transcription via PhoP and PmrA, reducing LPS positive charge. Such intermediate charge could enhance fitness in non‐selective environments, enabling strains to compete with other microbes or evade host immunity. Susceptibility might also re‐emerge if mutations in *phoP*, *phoQ*, *pmrA* or *pmrB* prove deleterious.

Cross‐sensitivity represents another critical factor. While the isolates studied exhibited defined MICs for polymyxins, clinical treatment histories—unavailable here—may involve β‐lactams or other antibiotics. Thus, these strains could retain susceptibility to alternative agents, underscoring the need for comprehensive antimicrobial stewardship to delay resistance fixation. Collectively, these dynamics emphasise the complex interplay between adaptive evolution, compensatory mechanisms and therapeutic practices in shaping resistance trajectories within healthcare ecosystems.

### 4.5 Final Considerations and Study Limitations

This study elucidates the evolutionary mechanisms underpinning polymyxin resistance in *K. pneumoniae* , emphasising the interplay of positive selection, polygenic adaptation, population expansion and selective sweeps. The dominance of *mgrB* insertions in resistant populations underscores their central role in resistance via LPS modification. However, mutations in *phoPQ* and *pmrAB* also contribute to the phenotype, illustrating a polygenic architecture where multiple loci collectively drive adaptation. The prevalence of rare alleles and significantly negative Tajima's D and Fu & Li's values in both PM‐S and PM‐R suggest recent expansions temporally aligned with the resurgence of polymyxin use post‐2010. In PM‐S, the ancestral haplotype persists alongside emerging haplotypes, while in PM‐R, haplotype 27 is overrepresented. Haplotypes derived from haplotype 27 exhibit mutations in other loci, implying compensatory evolution to mitigate fitness costs, which could sustain genetic diversity despite strong selection.

The rapid clonal spread of haplotype 27, facilitated by horizontal gene transfer, exemplifies a classic hard sweep. However, incomplete fixation and persistent diversity point to soft sweeps or polygenic adaptation, where multiple mutations sustain resistance without eliminating variation. This complexity complicates containment strategies, as resistance may arise through diverse pathways.

These findings carry profound implications for global health. Without novel therapies, reliance on polymyxins will fuel further resistance, particularly in endemic regions. Compensatory mutations, while stabilising bacterial fitness, may indirectly prolong resistance persistence even in low‐drug‐pressure environments. Several study limitations warrant acknowledgment. The analysed population is historical rather than natural, with overrepresentation from Brazil and Portugal and limited European allele diversity. Additionally, *mcr* genes—though globally rare—were not assessed but may exist in the strains used in the study.

Future studies should expand genomic analyses to include *arn* and *pmrC* operons, given their influence on resistance. Experimental validation via gene‐edited mutants is essential to confirm compensatory mutations or hitchhiking effects in *phoPQ* and *pmrAB*. Addressing these gaps will refine our understanding of resistance dynamics and inform strategies to mitigate its evolution in clinical settings.

## Author Contributions

This study was designed by Daniel Miceli Serwy, Fabio Faria da Mota, Teca Calcagno Galvao and Viviane Zahner. Daniel Miceli Serwy conducted the experimental work. Data analysis was performed by Daniel Miceli Serwy, Ana Luiza Carneiro Alencar, Roberto Leonan Morim Novaes and Josué da Costa Lima‐Junior. Ana Paula Carvalho‐Assef and Maria Eduarda Rocha Conde designed and carried out MIC testing, respectively. The manuscript was written by Daniel Miceli Serwy, Teca Calcagno Galvao and Viviane Zahner.

## Funding

Daniel Serwy's Masters program scholarship was funded by CAPES (Foundation for the Coordination of Improvement of Higher Education Personnel, Education Ministry/MEC). The project was funded by Conselho Nacional de Desenvolvimento Científico e Tecnológico (CNPq grant 421136/2023‐5), Fundação Carlos Chagas Filho de Amparo à Pesquisa do Estado do Rio de Janeiro (FAPERJ grants E‐26/210.228/2018 and 11E‐26/210.982/2021) and Fiocruz.

## Conflicts of Interest

The authors declare no conflicts of interest.

## Supporting information


**Data S1:** mec70234‐sup‐0001‐DataS1.docx.

**Table S1:** mec70234‐sup‐0002‐TableS1.docx.

**Table S2:** mec70234‐sup‐0003‐TableS2.docx.

**Table S3:** mec70234‐sup‐0004‐TableS3.docx.

## Data Availability

The data reported in this manuscript is available in its body as well as in Supporting Information. The authors remain available for further enquiries and/or doubts. WGS sequences can be accessed from the National Center for Biotechnology Information (NCBI) via BioProjects PRJNA307517, PRJEB38289, PRJNA385863 and PRJNA316321.

## References

[mec70234-bib-0001] Arcari, G. , and A. Carattoli . 2022. “Global Spread and Evolutionary Convergence of Multidrug‐Resistant and Hypervirulent *Klebsiella pneumoniae* High‐Risk Clones.” Pathogens and Global Health 117, no. 4: 328–341. 36089853 10.1080/20477724.2022.2121362 PMC10177687

[mec70234-bib-0002] Bandelt, H. J. , P. Forster , and A. Röhl . 1999. “Median‐Joining Networks for Inferring Intraspecific Phylogenies.” Molecular Biology and Evolution 16, no. 1: 37–48. 10331250 10.1093/oxfordjournals.molbev.a026036

[mec70234-bib-0003] Booker, T. R. , B. C. Jackson , and P. D. Keightley . 2017. “Detecting Positive Selection in the Genome.” BMC Biology 15, no. 1: 98. 29084517 10.1186/s12915-017-0434-y PMC5662103

[mec70234-bib-0004] BrCAST (Brazilian Committee on Antimicrobial Susceptibility Testing) . 2023. “Recommendations for Antimicrobial Susceptibility Testing (AST).” https://brcast.org.br/documentos.

[mec70234-bib-0005] Budia‐Silva, M. , T. Kostyanev , S. Ayala‐Montaño , et al. 2024. “International and Regional Spread of Carbapenem‐Resistant *Klebsiella pneumoniae* in Europe.” Nature Communications 15: 5092. 10.1038/s41467-024-49349-z PMC11178878 38877000

[mec70234-bib-0006] Chen, S. , Y. Zhou , Y. Chen , and J. Gu . 2018. “Fastp: An Ultra‐Fast All‐In‐One FASTQ Preprocessor.” Bioinformatics 34, no. 17: i884–i890. 30423086 10.1093/bioinformatics/bty560 PMC6129281

[mec70234-bib-0007] CLSI (Clinical and Laboratory Standards Institute) . 2023. Performance Standards for Antimicrobial Susceptibility Testing. 33rd ed. CLSI Supplement M100.

[mec70234-bib-0008] Conceição‐Neto, O. C. , B. S. da Costa , L. D. S. Pontes , et al. 2022. “Polymyxin Resistance in Clinical Isolates of *K. pneumoniae* in Brazil: Update on Molecular Mechanisms, Clonal Dissemination and Relationship With KPC‐Producing Strains.” Frontiers in Cellular and Infection Microbiology 12: 898125. 35909953 10.3389/fcimb.2022.898125 PMC9334684

[mec70234-bib-0009] Danecek, P. , J. K. Bonfield , J. Liddle , et al. 2021. “Twelve Years of SAMtools and BCFtools.” GigaScience 10, no. 2: giab008. 33590861 10.1093/gigascience/giab008 PMC7931819

[mec70234-bib-0010] Diancourt, L. , V. Passet , J. Verhoef , P. A. Grimont , and S. Brisse . 2005. “Multilocus Sequence Typing of *Klebsiella pneumoniae* Nosocomial Isolates.” Journal of Clinical Microbiology 43: 4178–4182. 16081970 10.1128/JCM.43.8.4178-4182.2005 PMC1233940

[mec70234-bib-0011] Eldholm, V. , G. Norheim , B. von der Lippe , et al. 2014. “Evolution of Extensively Drug‐Resistant *Mycobacterium tuberculosis* From a Susceptible Ancestor in a Single Patient.” Genome Biology 15, no. 11: 490. 25418686 10.1186/s13059-014-0490-3 PMC4223161

[mec70234-bib-0012] Elias, R. , A. Spadar , J. Phelan , et al. 2022. “A Phylogenomic Approach for the Analysis of Colistin Resistance‐Associated Genes in *Klebsiella pneumoniae* , Its Mutational Diversity and Implications for Phenotypic Resistance.” International Journal of Antimicrobial Agents 59, no. 6: 106581. 35378228 10.1016/j.ijantimicag.2022.106581

[mec70234-bib-0013] El‐Sayed Ahmed, M. A. , L. L. Zhong , C. Shen , Y. Yang , Y. Doi , and G. B. Tian . 2020. “Colistin and Its Role in the Era of Antibiotic Resistance: An Extended Review (2000–2019).” Emerging Microbes & Infections 9, no. 1: 868–885. 32284036 10.1080/22221751.2020.1754133 PMC7241451

[mec70234-bib-0014] EUCAST (European Committee on Antimicrobial Susceptibility Testing) . 2023. “Breakpoint Tables for Interpretation of MICs and Zone Diameters.” http://www.eucast.org.

[mec70234-bib-0015] Ferretti, L. , A. Ledda , T. Wiehe , G. Achaz , and S. E. Ramos‐Onsins . 2017. “Decomposing the Site Frequency Spectrum: The Impact of Tree Topology on Neutrality Tests.” Genetics 207: 229–240. 28679545 10.1534/genetics.116.188763 PMC5586374

[mec70234-bib-0016] Fisher, R. A. 1918. “The Correlation Between Relatives on the Supposition of Mendelian Inheritance.” Transactions of the Royal Society of Edinburgh 53: 399–433.

[mec70234-bib-0017] Fleming, A. 1945. Penicillin's Therapeutic Potential and the Rise of Resistance. Nobel Lecture.

[mec70234-bib-0018] Fluxus Technology Ltd . 2023. “Network 10.2.” http://www.fluxus‐engineering.com.

[mec70234-bib-0019] Fu, Y. X. , and W. H. Li . 1993. “Statistical Tests of Neutrality of Mutations.” Genetics 133, no. 3: 693–709. 8454210 10.1093/genetics/133.3.693 PMC1205353

[mec70234-bib-0020] Hernando‐Amado, S. , T. M. Coque , F. Baquero , and J. L. Martínez . 2019. “Antibiotic Resistance: Moving From Individual Health Norms to Social Norms in One Health and Global Health.” Frontiers in Microbiology 10: 1914. 32983000 10.3389/fmicb.2020.01914 PMC7483582

[mec70234-bib-0021] Janssen, A. B. , D. J. Doorduijn , G. Mills , et al. 2020. “Evolution of Colistin Resistance in the *Klebsiella pneumoniae* Complex Follows Multiple Evolutionary Trajectories With Variable Effects on Fitness and Virulence Characteristics.” Antimicrobial Agents and Chemotherapy 65, no. 1: e01958‐20. 33139278 10.1128/AAC.01958-20 PMC7927857

[mec70234-bib-0022] Jayol, A. , L. Poirel , A. Brink , M. V. Villegas , M. Yilmaz , and P. Nordmann . 2014. “Resistance to Colistin Associated With a Single Amino Acid Change in Protein PmrB Among *Klebsiella pneumoniae* Isolates of Worldwide Origin.” Antimicrobial Agents and Chemotherapy 58, no. 8: 4762–4766. 24914122 10.1128/AAC.00084-14 PMC4136042

[mec70234-bib-0023] Kearse, M. , R. Moir , and A. Wilson . 2012. “Geneious Basic: An Integrated and Extendable Desktop Software Platform for the Organization and Analysis of Sequence Data.” Bioinformatics 28, no. 12: 1647–1649. 22543367 10.1093/bioinformatics/bts199 PMC3371832

[mec70234-bib-0024] Kumar, S. , G. Stecher , M. Li , C. Knyaz , and K. Tamura . 2021. “MEGA11: Molecular Evolutionary Genetics Analysis Version 11.” Molecular Biology and Evolution 38, no. 7: 3022–3027. 33892491 10.1093/molbev/msab120 PMC8233496

[mec70234-bib-0025] Lam, M. M. C. , R. R. Wick , S. C. Watts , et al. 2021. “A Genomic Surveillance Framework and Genotyping Tool for *Klebsiella pneumoniae* and Its Related Species Complex.” Nature Communications 12: 4188. 10.1038/s41467-021-24448-3 PMC8263825 34234121

[mec70234-bib-0026] Landman, D. , C. Georgescu , D. A. Martin , and J. Quale . 2008. “Polymyxins Revisited.” Clinical Microbiology Reviews 21, no. 3: 449–465. 18625681 10.1128/CMR.00006-08 PMC2493081

[mec70234-bib-0027] Leinonen, R. , H. Sugawara , and M. Shumway . 2011. “The Sequence Read Archive.” Nucleic Acids Research 39: D19–D21. 21062823 10.1093/nar/gkq1019 PMC3013647

[mec70234-bib-0028] Levin, B. R. , V. Perrot , and N. Walker . 2000. “Compensatory Mutations, Antibiotic Resistance and the Population Genetics of Adaptive Evolution in Bacteria.” Genetics 154, no. 3: 985–997. 10757748 10.1093/genetics/154.3.985 PMC1460977

[mec70234-bib-0029] Li, H. 2013. “Aligning Sequence Reads, Clone Sequences, and Assembly Contigs With BWA‐MEM.” Preprint, arRxiv, March 16. https://arxiv.org/abs/1303.3997.

[mec70234-bib-0030] Lourenço, A. T. O. 2021. Identificação de marcadores moleculares preditivos de sensibilidade/resistência à colistina em *Klebsiella pneumoniae* , 243. Fundação Pio XII—Hospital de Câncer de Barretos.

[mec70234-bib-0031] Maynard Smith, J. , and J. Haigh . 1974. “The Hitch‐Hiking Effect of a Favourable Gene.” Genetics Research 23, no. 1: 23–35. 4407212

[mec70234-bib-0032] Moffatt, J. H. , M. Harper , P. Harrison , et al. 2010. “Colistin Resistance in *Klebsiella pneumoniae* : Molecular Mechanisms and Strain Epidemiology.” Antimicrobial Agents and Chemotherapy 54, no. 12: 4971–4977. 20855724 10.1128/AAC.00834-10 PMC2981238

[mec70234-bib-0033] Munoz‐Price, L. S. , L. Poirel , R. A. Bonomo , et al. 2013. “Clinical Epidemiology of the Global Expansion of *Klebsiella pneumoniae* Carbapenemases.” Lancet Infectious Diseases 13, no. 9: 785–796. 23969216 10.1016/S1473-3099(13)70190-7 PMC4673667

[mec70234-bib-0034] Nation, R. L. , T. Velkov , and J. Li . 2014. “Colistin and Polymyxin B: Peas in a Pod, or Chalk and Cheese?” Clinical Infectious Diseases 59, no. 1: 88–94. 24700659 10.1093/cid/ciu213 PMC4305129

[mec70234-bib-0035] NCBI Resource Coordinators . 2018. “Database Resources of the National Center for Biotechnology Information.” Nucleic Acids Research 46, no. D1: D8–D13. 29140470 10.1093/nar/gkx1095 PMC5753372

[mec70234-bib-0036] Nordmann, P. , T. Naas , and L. Poirel . 2011. “The Real Threat of *Klebsiella pneumoniae* Carbapenemase‐Producing Bacteria.” Lancet Infectious Diseases 11, no. 1: 22–31. 10.1016/S1473-3099(09)70054-4 19324295

[mec70234-bib-0037] Olaitan, A. O. , S. Morand , and J. M. Rolain . 2014. “Mechanisms of Polymyxin Resistance: Acquired and Intrinsic Resistance in Bacteria.” Frontiers in Microbiology 5: 643. 25505462 10.3389/fmicb.2014.00643 PMC4244539

[mec70234-bib-0038] O'Neill, J. 2016. “Tackling Drug‐Resistant Infections Globally: Final Report and Recommendations.” In The Review on Antimicrobial Resistance, 84. Wellcome Trust. https://amr‐review.org.

[mec70234-bib-0039] Partridge, S. R. , S. M. Kwong , N. Firth , and S. O. Jensen . 2018. “Mobile Genetic Elements Associated With Antimicrobial Resistance.” Clinical Microbiology Reviews 31, no. 4: e00088‐17. 30068738 10.1128/CMR.00088-17 PMC6148190

[mec70234-bib-0040] Paterson, D. L. , and R. A. Bonomo . 2005. “Extended‐Spectrum β‐Lactamases: A Clinical Update.” Clinical Microbiology Reviews 18, no. 4: 657–686. 16223952 10.1128/CMR.18.4.657-686.2005 PMC1265908

[mec70234-bib-0041] Pitt, M. E. , A. G. Elliott , M. D. Cao , et al. 2018. “Multifactorial Chromosomal Variants Regulate Polymyxin Resistance in Extensively Drug‐Resistant *Klebsiella pneumoniae* .” Microbial Genomics 4, no. 3: e000158. 29431605 10.1099/mgen.0.000158 PMC5885010

[mec70234-bib-0042] Podolsky, S. H. 2015. “The Evolving Response to Antibiotic Resistance (1945–2018).” Perspectives in Biology and Medicine 58, no. 1: 67–78.

[mec70234-bib-0043] Podschun, R. , and U. Ullmann . 1998. “ *Klebsiella* spp. as Nosocomial Pathogens: Epidemiology, Taxonomy, Typing Methods, and Pathogenicity Factors.” Clinical Microbiology Reviews 11, no. 4: 589–603. 9767057 10.1128/cmr.11.4.589 PMC88898

[mec70234-bib-0044] Poirel, L. , A. Jayol , S. Bontron , et al. 2015. “The *mgrB* Gene as a Key Target for Acquired Resistance to Colistin in *Klebsiella pneumoniae* .” Journal of Antimicrobial Chemotherapy 70, no. 1: 75–80. 25190723 10.1093/jac/dku323

[mec70234-bib-0045] Poirel, L. , A. Jayol , and P. Nordmann . 2015. “Polymyxins: Antibacterial Activity, Susceptibility Testing, and Resistance Mechanisms Encoded by Plasmids or Chromosomes.” Clinical Microbiology Reviews 28, no. 2: 557–596. 10.1128/CMR.00064-16 PMC5355641 28275006

[mec70234-bib-0046] Pritchard, J. K. , J. K. Pickrell , and G. Coop . 2010. “The Genetics of Human Adaptation: Hard Sweeps, Soft Sweeps, and Polygenic Adaptation.” Current Biology 20, no. 4: R208–R215. 20178769 10.1016/j.cub.2009.11.055 PMC2994553

[mec70234-bib-0047] Quinlan, A. R. , and I. M. Hall . 2010. “BEDTools: A Flexible Suite of Utilities for Comparing Genomic Features.” Bioinformatics 26, no. 6: 841–842. 20110278 10.1093/bioinformatics/btq033 PMC2832824

[mec70234-bib-0048] Rimoldi, S. G. , B. Gentile , C. Pagani , et al. 2017. “Whole Genome Sequencing for the Molecular Characterization of Carbapenem‐Resistant *Klebsiella pneumoniae* Strains Isolated at the Italian ASST Fatebenefratelli Sacco Hospital, 2012–2014.” BMC Infectious Diseases 17, no. 1: 666. 29017452 10.1186/s12879-017-2760-7 PMC5634883

[mec70234-bib-0049] Rodrigues, C. , S. Desai , V. Passet , D. Gajjar , and S. Brisse . 2022. “Genomic Evolution of the Globally Disseminated Multidrug‐Resistant *Klebsiella pneumoniae* Clonal Group 147.” Microbial Genomics 8: e000737. 10.1099/mgen.0.000737 PMC8914359 35019836

[mec70234-bib-0050] Rozas, J. , A. Ferrer‐Mata , J. C. Sánchez‐DelBarrio , et al. 2017. “DnaSP 6: DNA Sequence Polymorphism Analysis of Large Data Sets.” Molecular Biology and Evolution 34, no. 12: 3299–3302. 29029172 10.1093/molbev/msx248

[mec70234-bib-0051] Sahoo, S. , R. K. Sahoo , S. Dixit , et al. 2023. “NDM‐5‐Carrying *Klebsiella pneumoniae* ST437 Belonging to High‐Risk Clonal Complex (CC11) From an Urban River in Eastern India.” 3 Biotech 13: 139. 10.1007/s13205-023-03556-5 PMC10133422 37124981

[mec70234-bib-0052] Sameni, F. , M. Ghazi , M. Dadashi , et al. 2022. “Global Distribution, Genotypes and Prevalent Sequence Types of Colistin‐Resistant *Klebsiella pneumoniae* Isolated From Clinical Samples; a Systematic Review.” Gene Reports 28: 101635.

[mec70234-bib-0053] Seemann, T. 2014. “Prokka: Rapid Prokaryotic Genome Annotation.” Bioinformatics 30, no. 14: 2068–2069. 24642063 10.1093/bioinformatics/btu153

[mec70234-bib-0054] Szijártó, V. , L. M. Guachalla , K. Hartl , et al. 2016. “Both Clades of the Epidemic KPC‐Producing *Klebsiella pneumoniae* Clone ST258 Share a Modified Galactan O‐Antigen Type.” International Journal of Medical Microbiology 306, no. 2: 89–98. 26723873 10.1016/j.ijmm.2015.12.002

[mec70234-bib-0055] Tajima, F. 1989. “Statistical Method for Testing the Neutral Mutation Hypothesis by DNA Polymorphism.” Genetics 123, no. 3: 585–595. 2513255 10.1093/genetics/123.3.585 PMC1203831

[mec70234-bib-0056] Velkov, T. , K. D. Roberts , R. L. Nation , P. E. Thompson , and J. Li . 2013. “Pharmacology of Polymyxins: New Insights Into an ‘Old’ Class of Antibiotics.” Future Microbiology 8, no. 6: 711–724. 23701329 10.2217/fmb.13.39 PMC3852176

[mec70234-bib-0057] Ventola, C. L. 2015. “The Antibiotic Resistance Crisis: Part 1: Causes and Threats.” Pharmacy and Therapeutics 40, no. 4: 277–283. 25859123 PMC4378521

[mec70234-bib-0058] Wilson, B. A. , N. R. Garud , A. F. Feder , Z. J. Assaf , and P. S. Pennings . 2016. “The Population Genetics of Drug Resistance Evolution in Natural Populations of Viral, Bacterial and Eukaryotic Pathogens.” Molecular Ecology 25, no. 1: 42–66. 26578204 10.1111/mec.13474 PMC4943078

[mec70234-bib-0059] World Health Organization . 2024. “WHO Bacterial Priority Pathogens List, 2024: Bacterial Pathogens of Public Health Importance to Guide Research, Development and Strategies to Prevent and Control Antimicrobial Resistance [Internet].” https://www.who.int/publications/i/item/9789240093461.

[mec70234-bib-0060] Wu, Y. , T. Jiang , X. He , et al. 2023. “Global Phylogeography and Genomic Epidemiology of Carbapenem‐Resistant Blaoxa‐232–Carrying *Klebsiella pneumoniae* Sequence Type 15 Lineage.” Emerging Infectious Diseases 29, no. 11: 2246–2256. 37877525 10.3201/eid2911.230463 PMC10617323

[mec70234-bib-0061] Wyres, K. L. , and K. E. Holt . 2018. “ *Klebsiella pneumoniae* Population Genomics and Antimicrobial‐Resistant Clones.” Trends in Microbiology 26, no. 12: 944–956. 10.1016/j.tim.2016.09.007 27742466

[mec70234-bib-0062] Wyres, K. L. , M. M. C. Lam , and K. E. Holt . 2020. “Population Genomics of *Klebsiella pneumoniae* .” Nature Reviews Microbiology 18: 344–359. 32055025 10.1038/s41579-019-0315-1

[mec70234-bib-0063] Zhu, Y. , I. Galani , I. Karaiskos , et al. 2019. “Multifaceted Mechanisms of Colistin Resistance Revealed by Genomic Analysis of Multidrug‐Resistant *Klebsiella pneumoniae* Isolates From Individual Patients Before and After Colistin Treatment.” Journal of Infection 79, no. 4: 312–321. 31374222 10.1016/j.jinf.2019.07.009 PMC7264071

