# Supplementary material for: Genetic Diversity of Polymyxin Resistance Genes in Klebsiella pneumoniae Clinical Isolates
Source: Mol Ecol. 2026 Jan 20;35(2):e70234. doi: 10.1111/mec.70234 (PMC12817155; doi:10.1111/mec.70234)
Supplement: Supplementary file 1 — Data S1: mec70234‐sup‐0001‐DataS1.docx. [file MEC-35-e70234-s004.docx]

| **Table 1**: PCR Reaction | | |
| --- | --- | --- |
| **Component** | **Volume** | **Final Concentration** |
| GoTaq® Green Master Mix, 2X | 12.5 µl | 1X |
| Upstream primer, 10µM | 1,25 µl | 0,5 µM |
| Downstream primer, 10µM | 1,25 µl | 0,5 µM |
| DNA template | 5 µl | 10 ng |
| Nuclease-Free Water | 5 µl | N.A. |

25 µl

| **Table 2**: Genes & amplicon size | | |
| --- | --- | --- |
| **Gene** | **Gene size (bp)** | **Amplicon Size (bp)** |
| *mgrB* | 147 | 450 |
| *phoP* | 671 | 739 |
| *phoQ* | 1466 | 1596 |
| *pmrA* | 671 | 850 |
| *pmrB* | 1097 | 1273 |

| **Table 3**: Primer Sequences | | | |
| --- | --- | --- | --- |
| **Primer** | **Sequence** | **Type** | **Primer Design** |
| *mgrB* ext F2 | TGGCACTTAAGACCCAGACC | External Foward (Promoter region) | This Study (Teca Galvão) |
| *phoQ* int F | CAGCAAAATGCCTCAGCT | Internal Foward | Jayol *et al*., 2014 |
| *pmrB* int F | CGCCGACGTGGCGCATGA | Internal Foward | Jayol *et al*., 2014 |
| *pmrA* ext F | CATTTCCGCGCACTGTCTGC | External Foward | Jayol *et al*., 2014 |
| *pmrA* ext R | CAGGTTTCAGTTGCAAACAG | External Reverse | Jayol *et al*., 2014 |
| *pmrB* ext F | ACCTACGCGAAAAGATTGGC | External Foward | Jayol *et al*., 2014 |
| *pmrB* ext R | GATGAGGATAGCGCCCATGC | External Reverse | Jayol *et al*., 2014 |
| *phoP* ext F | GAGCTTCAGACTACTATCGA | External Foward | Jayol *et al*., 2014 |
| *phoP* ext R | GGGAAGATATGCCGCAACAG | External Reverse | Jayol *et al*., 2014 |
| *phoQ* ext F | ATACCCACAGGACGTCATCA | External Foward | Jayol *et al*., 2014 |
| *phoQ* ext R | CAGGTGTCTGACAGGGATTA | External Reverse | Jayol *et al*., 2014 |
| *mgrB* ext F | TTAAGAAGGCCGTGCTATCC | External Foward | Cannatelli *et al*., 2013 |
| *mgrB* ext R | AAGGCGTTCATTCTACCACC | External Reverse | Cannatelli *et al*., 2013 |

| **Table 4**: PCR parameters | | | |
| --- | --- | --- | --- |
| *mgrB* |  |  |  |
| **Stage** | **Temperature (°C)** | **Time** | **Cycles** |
| Initial denaturation | 95 ºC | 2 minutes | 1 |
| Denaturation | 95 ºC | 30 seconds | 35 |
| Annealing | 54 ºC | 30 seconds |  |
| Extension | 72 ºC | 2 minutes |  |
| Final extension | 72 ºC | 5 minutes | 1 |
|  |  |  |  |
|  |  |  |  |
| *phoP* |  |  |  |
| **Stage** | **Temperature (°C)** | **Time** | **Cycles** |
| Initial denaturation | 95 ºC | 2 minutes | 1 |
| Denaturation | 95 ºC | 30 seconds | 35 |
| Annealing | 49 ºC | 30 seconds |  |
| Extension | 72 ºC | 1 minute |  |
| Final extension | 72 ºC | 5 minutes | 1 |
|  |  |  |  |
| *phoQ* |  |  |  |
| **Stage** | **Temperature (°C)** | **Time** | **Cycles** |
| Initial denaturation | 95 ºC | 2 minutes | 1 |
| Denaturation | 95 ºC | 30 seconds | 35 |
| Annealing | 53 ºC | 30 seconds |  |
| Extension | 72 ºC | 2 minutes |  |
| Final extension | 72 ºC | 5 minutes | 1 |
|  |  |  |  |
| *pmrA* |  |  |  |
| **Stage** | **Temperature (°C)** | **Time** | **Cycles** |
| Initial denaturation | 95 ºC | 2 minutes | 1 |
| Denaturation | 95 ºC | 30 seconds | 35 |
| Annealing | 50 ºC | 30 seconds |  |
| Extension | 72 ºC | 1 minute |  |
| Final extension | 72 ºC | 5 minutes | 1 |
|  |  |  |  |
| *pmrB* |  |  |  |
| **Stage** | **Temperature (°C)** | **Time** | **Cycles** |
| Initial denaturation | 95 ºC | 2 minutes | 1 |
| Denaturation | 95 ºC | 30 seconds | 35 |
| Annealing | 53 ºC | 30 seconds |  |
| Extension | 72 ºC | 2 minutes |  |
| Final extension | 72 ºC | 5 minutes | 1 |
