# Supplementary material for: Genetic Diversity of Polymyxin Resistance Genes in Klebsiella pneumoniae Clinical Isolates
Source: Mol Ecol. 2026 Jan 20;35(2):e70234. doi: 10.1111/mec.70234 (PMC12817155; doi:10.1111/mec.70234)
Supplement: Supplementary file 2 — Table S1: mec70234‐sup‐0002‐TableS1.docx. [file MEC-35-e70234-s002.docx]

| **Table S1**: QC metrics | | | |
| --- | --- | --- | --- |
| **SRA Accession** | **Coverage** | **Assembly Accession** | **Coverage2** |
| Average | 91,21557045 | Average | 102,2905 |
| Standard deviation | 22,6767221 | Standard deviation | 82,01877782 |
| ERR10030668_1 | 95,8918 | GCF_001701355.1 | 100 |
| ERR10030668_2 | 92,7573 | GCF_001701365.1 | 100 |
| ERR10030671_1 | 86,0194 | GCF_001701445.1 | 100 |
| ERR10030675_1 | 94,177 | GCF_001701505.1 | 100 |
| ERR10030675_2 | 88,0089 | GCF_001701525.1 | 100 |
| ERR10030682_1 | 83,3964 | GCF_001701555.1 | 100 |
| ERR10030696_1 | 79,3539 | GCF_001701895.1 | 100 |
| ERR10030696_2 | 75,1837 | GCF_001701925.1 | 100 |
| ERR10030697_2 | 85,1978 | GCF_001701975.1 | 100 |
| ERR10030700_1 | 82,5589 | GCF_001702035.1 | 100 |
| ERR10030700_2 | 76,7399 | GCF_002197505.1 | 85 |
| ERR10030701_1 | 87,6342 | GCF_002235325.1 | 355 |
| ERR10030701_2 | 84,2187 | GCF_002235355.1 | 311 |
| ERR10030704_2 | 82,3892 | GCF_002235475.1 | 199 |
| ERR10030705_1 | 80,2552 | GCF_002235535.1 | 275 |
| ERR10030705_2 | 75,6853 | GCF_002235895.1 | 127 |
| ERR10030707_2 | 72,0524 | GCF_002236275.1 | 162 |
| ERR10030710_1 | 90,8352 | GCF_002236305.1 | 228 |
| ERR10030710_2 | 88,8143 | GCF_002968475.1 | 16,41 |
| ERR10030711_2 | 83,1827 | GCF_003324315.1 | 16,21 |
| ERR10030717_2 | 53,7503 | GCA_903171855.1 | 150 |
| ERR10030718_2 | 95,9769 | GCF_903171865.1 | 150 |
| ERR10030720_1 | 69,8382 | GCF_903171875.1 | 150 |
| ERR10030720_2 | 67,2122 | GCF_903171895.1 | 150 |
| ERR10030722_1 | 69,9149 | GCF_903171885.1 | 150 |
| ERR10030728_1 | 67,041 | GCF_903171925.1 | 150 |
| ERR10030728_2 | 61,5572 | GCF_007655375.1 | 31 |
| ERR10030729_2 | 69,8975 | GCF_007655395.1 | 27 |
| ERR10030730_1 | 124,119 | GCF_007655405.1 | 30 |
| ERR10030730_2 | 118,239 | GCF_007655445.1 | 33 |
| ERR10030731_1 | 69,968 | GCF_007655475.1 | 36 |
| ERR10030731_2 | 61,8985 | GCF_007655535.1 | 34 |
| ERR10030732_1 | 103,983 | GCF_007657875.1 | 27 |
| ERR10030732_2 | 101,819 | GCF_007657885.1 | 27 |
| ERR10030733_1 | 114,695 | GCF_007657905.1 | 29 |
| ERR10030738_2 | 94,9064 | GCF_007657915.1 | 30 |
| ERR10030739_1 | 96,4909 | GCF_007657925.1 | 29 |
| ERR10030739_2 | 95,9104 | GCF_007657945.1 | 28 |
| ERR10030741_1 | 122,475 | GCF_007657965.1 | 26 |
| ERR10030741_2 | 121,051 | GCF_007658005.1 | 30 |
| ERR10030743_1 | 74,4785 |  |  |
| ERR10030743_2 | 72,752 |  |  |
| ERR10030744_1 | 106,757 |  |  |
| ERR10030746_1 | 93,2224 |  |  |
| ERR10030746_2 | 91,107 |  |  |
| ERR10030747_1 | 100,626 |  |  |
| ERR10030748_1 | 99,1529 |  |  |
| ERR10030748_2 | 95,4517 |  |  |
| ERR10030749_1 | 87,7883 |  |  |
| ERR10030749_2 | 85,5726 |  |  |
| ERR10030750_1 | 98,925 |  |  |
| ERR10030757_1 | 88,9863 |  |  |
| ERR10030757_2 | 86,0707 |  |  |
| ERR10030758_2 | 97,2971 |  |  |
| ERR10030759_2 | 81,1323 |  |  |
| ERR10030762_1 | 109,283 |  |  |
| ERR10030762_2 | 105,294 |  |  |
| ERR10030781_1 | 86,6041 |  |  |
| ERR10030799_1 | 95,9233 |  |  |
| ERR10030820_1 | 112,503 |  |  |
| ERR10030820_2 | 112,245 |  |  |
| ERR10030821_1 | 99,1005 |  |  |
| ERR10030822_1 | 93,1445 |  |  |
| ERR10030822_2 | 92,001 |  |  |
| ERR10030824_1 | 97,7669 |  |  |
| ERR10030824_2 | 96,7717 |  |  |
| ERR10030826_1 | 99,071 |  |  |
| ERR10030827_2 | 88,4577 |  |  |
| ERR10030862_2 | 113,229 |  |  |
| ERR10030863_2 | 88,2214 |  |  |
| ERR10030864_1 | 111,498 |  |  |
| ERR10030865_2 | 98,9275 |  |  |
| ERR10030866_2 | 0,856292 |  |  |
| ERR10030867_2 | 81,3559 |  |  |
| ERR10030868_1 | 93,2825 |  |  |
| ERR10030869_1 | 94,5493 |  |  |
| ERR10030873_1 | 84,1631 |  |  |
| ERR10030874_1 | 151,375 |  |  |
| ERR10030896_2 | 80,374 |  |  |
| ERR10030897_1 | 96,0884 |  |  |
| ERR10030897_2 | 95,3182 |  |  |
| ERR10030898_1 | 91,8876 |  |  |
| ERR10030898_2 | 90,9394 |  |  |
| ERR10030899_1 | 79,3341 |  |  |
| ERR10030899_2 | 78,8228 |  |  |
| ERR10030915_1 | 91,7967 |  |  |
| ERR10030917_2 | 84,1248 |  |  |
| ERR10030921_1 | 94,0541 |  |  |
| ERR10030925_2 | 104,633 |  |  |
| ERR10030963_1 | 117,848 |  |  |
| ERR10030972_1 | 101,51 |  |  |
| ERR10030972_2 | 100,881 |  |  |
| ERR10030973_2 | 104,003 |  |  |
| ERR10030976_1 | 62,5547 |  |  |
| ERR10030977_1 | 104,264 |  |  |
| ERR10030997_2 | 68,7717 |  |  |
| ERR10031001_1 | 102,222 |  |  |
| ERR10031001_2 | 100,419 |  |  |
| ERR10031017_1 | 111,689 |  |  |
| ERR10031017_2 | 111,718 |  |  |
| ERR10031020_1 | 116,216 |  |  |
| ERR10031020_2 | 114,274 |  |  |
| ERR10031021_1 | 92,9188 |  |  |
| ERR10031021_2 | 92,0978 |  |  |
| ERR10031134_1 | 92,4334 |  |  |
| ERR10031135_1 | 112,757 |  |  |
| ERR10031135_2 | 109,426 |  |  |
| ERR10031137_2 | 102,652 |  |  |
| ERR10031138_2 | 93,244 |  |  |
| ERR10031140_1 | 122,943 |  |  |
| ERR10031141_1 | 103,35 |  |  |
| ERR10031143_1 | 116,833 |  |  |
| ERR10031144_1 | 94,3083 |  |  |
| ERR10031144_2 | 91,9327 |  |  |
| ERR10031145_1 | 98,4552 |  |  |
| ERR10031145_2 | 95,7146 |  |  |
| ERR10031146_1 | 65,5133 |  |  |
| ERR2743752_1 | 13,6749 |  |  |
| ERR2743752_2 | 13,3562 |  |  |
| SRR4025984_2 | 28,3609 |  |  |
| SRR5168387_1 | 38,6394 |  |  |
| SRR5168388_2 | 39,5525 |  |  |
| SRR6799394_2 | 127,099 |  |  |
| SRR8607451_1 | 125,24 |  |  |
| SRR8607451_2 | 137,561 |  |  |
| SRR8607454_1 | 100,028 |  |  |
| SRR8607454_2 | 98,2313 |  |  |
| SRR8607455_2 | 132,332 |  |  |
| SRR8607456_1 | 130,918 |  |  |
| SRR8607456_2 | 131,346 |  |  |
| SRR8607457_1 | 101,277 |  |  |
| SRR8607461_1 | 73,6672 |  |  |
| SRR8607461_2 | 79,9069 |  |  |
| SRR8607463_1 | 103,41 |  |  |
| SRR8607463_2 | 107,177 |  |  |
| SRR8607464_1 | 73,4532 |  |  |
| SRR8607466_2 | 80,9834 |  |  |
| SRR8607471_1 | 58,213 |  |  |
| SRR8607471_2 | 66,1757 |  |  |
