# Supplementary material for: Genetic Diversity of Polymyxin Resistance Genes in Klebsiella pneumoniae Clinical Isolates
Source: Mol Ecol. 2026 Jan 20;35(2):e70234. doi: 10.1111/mec.70234 (PMC12817155; doi:10.1111/mec.70234)
Supplement: Supplementary file 3 — Table S2: mec70234‐sup‐0003‐TableS2.docx. [file MEC-35-e70234-s001.docx]

| **Table S2**: *rpoB* alleles and MLST diversity | | | | | |
| --- | --- | --- | --- | --- | --- |
| **Strain** | **MIC** | **Phenotype** | **Country** | ***rpoB* Allele** | **ST** |
| ATH15 | 0,125 | Susceptible | Greece | 1 | 258 |
| Kp1003 | 0,25 | Susceptible | Portugal | 1 | 11 |
| Kp3725 | 0,25 | Susceptible | Portugal | 1 | 11 |
| Kp828 | 0,25 | Susceptible | Portugal | 1 | 12 |
| Kp829 | 0,25 | Susceptible | Portugal | 1 | 12 |
| Kp4279 | 0,25 | Susceptible | Portugal | 1 | 13 |
| Kp748 | 0,25 | Susceptible | Portugal | 1 | 14 |
| Kp850 | 0,25 | Susceptible | Portugal | 1 | 15 |
| Kp874 | 0,25 | Susceptible | Portugal | 1 | 15 |
| Kp875 | 0,25 | Susceptible | Portugal | 1 | 15 |
| Kp888 | 0,25 | Susceptible | Portugal | 1 | 15 |
| Kp1001 | 0,25 | Susceptible | Portugal | 1 | 15 |
| Kp1019 | 0,25 | Susceptible | Portugal | 1 | 15 |
| Kp910 | 0,25 | Susceptible | Portugal | 1 | 15 |
| Kp972 | 0,25 | Susceptible | Portugal | 1 | 15 |
| Kp986 | 0,25 | Susceptible | Portugal | 1 | 15 |
| Kp997 | 0,25 | Susceptible | Portugal | 1 | 15 |
| Kp1031 | 0,25 | Susceptible | Portugal | 1 | 15 |
| Kp1032 | 0,25 | Susceptible | Portugal | 1 | 15 |
| Kp1036 | 0,25 | Susceptible | Portugal | 1 | 15 |
| Kp1122 | 0,25 | Susceptible | Portugal | 1 | 15 |
| Kp1144 | 0,25 | Susceptible | Portugal | 1 | 15 |
| Kp1363 | 0,25 | Susceptible | Portugal | 1 | 15 |
| Kp4287 | 0,25 | Susceptible | Portugal | 4 | 25 |
| Kp4367 | 0,25 | Susceptible | Portugal | 4 | 25 |
| Kp4408 | 0,25 | Susceptible | Portugal | 4 | 25 |
| Kp684 | 0,25 | Susceptible | Portugal | 4 | 25 |
| Kp689 | 0,25 | Susceptible | Portugal | 4 | 25 |
| Kp1209 | 0,25 | Susceptible | Portugal | 1 | 35 |
| Kp4256 | 0,25 | Susceptible | Portugal | 1 | 37 |
| Kp4194 | 0,25 | Susceptible | Portugal | 1 | 37 |
| Kp1528 | 0,25 | Susceptible | Portugal | 1 | 39 |
| Kp776 | 0,25 | Susceptible | Portugal | 1 | 45 |
| Kp898 | 0,25 | Susceptible | Portugal | 1 | 45 |
| Kp1675 | 0,25 | Susceptible | Portugal | 1 | 48 |
| Kp4292 | 0,25 | Susceptible | Portugal | 10 | 70 |
| Kp2958 | 0,25 | Susceptible | Portugal | 1 | 76 |
| Kp2209 | 0,25 | Susceptible | Portugal | 1 | 133 |
| Kp1495 | 0,25 | Susceptible | Portugal | 4 | 147 |
| Kp4387 | 0,25 | Susceptible | Portugal | 25 | 158 |
| Kp4333 | 0,25 | Susceptible | Portugal | 1 | 252 |
| ATH17 | 0,25 | Susceptible | Greece | 1 | 258 |
| Kp3860 | 0,25 | Susceptible | Portugal | 1 | 307 |
| Kp2162 | 0,25 | Susceptible | Portugal | 4 | 336 |
| Kp3270 | 0,25 | Susceptible | Portugal | 15 | 348 |
| Kp4197 | 0,25 | Susceptible | Portugal | 1 | 1799 |
| Kp4246 | 0,5 | Susceptible | Portugal | 1 | 15 |
| ATH9 | 0,5 | Susceptible | Greece | 4 | 147 |
| kp36 | 0,5 | Susceptible | Italy | 1 | 258 |
| kp46 | 0,5 | Susceptible | Italy | 1 | 258 |
| kp49 | 0,5 | Susceptible | Italy | 1 | 258 |
| ATH21 | 0,5 | Susceptible | Greece | 1 | 258 |
| ATH23 | 0,5 | Susceptible | Greece | 1 | 258 |
| ATH25 | 0,5 | Susceptible | Greece | 1 | 258 |
| kp38 | 0,5 | Susceptible | Italy | 1 | 512 |
| Kp3323 | 1 | Susceptible | Portugal | 1 | 11 |
| Kp2564 | 1 | Susceptible | Portugal | 1 | 11 |
| Kp2476 | 1 | Susceptible | Portugal | 1 | 13 |
| Kp2645 | 1 | Susceptible | Portugal | 1 | 13 |
| Kp2948 | 1 | Susceptible | Portugal | 1 | 14 |
| Kp3046 | 1 | Susceptible | Portugal | 1 | 14 |
| Kp4195 | 1 | Susceptible | Portugal | 1 | 15 |
| Kp918 | 1 | Susceptible | Portugal | 1 | 15 |
| Kp919 | 1 | Susceptible | Portugal | 1 | 15 |
| Kp3185 | 1 | Susceptible | Portugal | 1 | 15 |
| Kp3509 | 1 | Susceptible | Portugal | 1 | 15 |
| Kp4869 | 1 | Susceptible | Portugal | 1 | 15 |
| Kp4857 | 1 | Susceptible | Portugal | 1 | 15 |
| Kp1938 | 1 | Susceptible | Portugal | 1 | 15 |
| Kp2287 | 1 | Susceptible | Portugal | 1 | 15 |
| Kp2334 | 1 | Susceptible | Portugal | 4 | 20 |
| Kp1990 | 1 | Susceptible | Portugal | 4 | 29 |
| Kp4864 | 1 | Susceptible | Portugal | 1 | 35 |
| Kp4855 | 1 | Susceptible | Portugal | 1 | 37 |
| Kp840 | 1 | Susceptible | Portugal | 1 | 43 |
| Kp3660 | 1 | Susceptible | Portugal | 10 | 70 |
| Kp4865 | 1 | Susceptible | Portugal | 10 | 70 |
| Kp1507 | 1 | Susceptible | Portugal | 1 | 133 |
| Kp2497 | 1 | Susceptible | Portugal | 1 | 134 |
| Kp4886 | 1 | Susceptible | Portugal | 4 | 147 |
| Kp4887 | 1 | Susceptible | Portugal | 4 | 147 |
| Kp2786 | 1 | Susceptible | Portugal | 4 | 152 |
| Kp2463 | 1 | Susceptible | Portugal | 4 | 218 |
| Kp2454 | 1 | Susceptible | Portugal | 1 | 231 |
| Kp3000 | 1 | Susceptible | Portugal | 1 | 231 |
| Kp4871 | 1 | Susceptible | Portugal | 1 | 307 |
| Kp4878 | 1 | Susceptible | Portugal | 1 | 307 |
| Kp2200 | 1 | Susceptible | Portugal | 4 | 336 |
| Kp2568 | 1 | Susceptible | Portugal | 4 | 336 |
| Kp2587 | 1 | Susceptible | Portugal | 4 | 336 |
| Kp2605 | 1 | Susceptible | Portugal | 4 | 336 |
| Kp2606 | 1 | Susceptible | Portugal | 4 | 336 |
| Kp4856 | 1 | Susceptible | Portugal | 15 | 348 |
| Kp4862 | 1 | Susceptible | Portugal | 15 | 348 |
| Kp2895 | 1 | Susceptible | Portugal | 1 | 726 |
| Kp2447 | 1 | Susceptible | Portugal | 4 | 730 |
| Kp2864 | 1 | Susceptible | Portugal | 5 | 1801 |
| Kp2224 | 1 | Susceptible | Portugal | 1 | 2176 |
| Kp4861 | 2 | Susceptible | Portugal | 4 | 17 |
| Kp4882 | 2 | Susceptible | Portugal | 4 | 147 |
| Kp4860 | 2 | Susceptible | Portugal | 1 | 307 |
| Kp1924 | 2 | Susceptible | Portugal | 4 | 336 |
| Kp3851 | 2 | Susceptible | Portugal | 15 | 348 |
| CCBH 23368 | 4 | Resistant | Brazil | 1 | 11 |
| 23_GR_12 | 8 | Resistant | Greece | 1 | 258 |
| Kp5505 | 16 | Resistant | Portugal | 1 | 13 |
| Kp5510 | 16 | Resistant | Portugal | 1 | 13 |
| Kp5508 | 16 | Resistant | Portugal | 1 | 13 |
| Kp5513 | 16 | Resistant | Portugal | 1 | 13 |
| Kp5514 | 16 | Resistant | Portugal | 1 | 13 |
| Kp5516 | 16 | Resistant | Portugal | 1 | 13 |
| Kp5520 | 16 | Resistant | Portugal | 1 | 13 |
| CCBH 22137 | 16 | Resistant | Brazil | 1 | 15 |
| CCBH 22408 | 16 | Resistant | Brazil | 1 | 15 |
| Kp5506 | 16 | Resistant | Portugal | 4 | 17 |
| Kp5509 | 16 | Resistant | Portugal | 4 | 17 |
| Kp5511 | 16 | Resistant | Portugal | 4 | 17 |
| Kp4164 | 16 | Resistant | Portugal | 4 | 20 |
| CCBH 23454 | 16 | Resistant | Brazil | 1 | 48 |
| CCBH 22997 | 16 | Resistant | Brazil | 4 | 147 |
| CCBH 23000 | 16 | Resistant | Brazil | 4 | 147 |
| 9_GR_12 | 16 | Resistant | Greece | 1 | 258 |
| 13_GR_14 | 16 | Resistant | Greece | 1 | 258 |
| Kp4889 | 16 | Resistant | Portugal | 1 | 307 |
| CCBH 22462 | 16 | Resistant | Brazil | 1 | 437 |
| Kp19 | 16 | Resistant | Italy | 1 | 512 |
| Kp34 | 16 | Resistant | Italy | 1 | 512 |
| Kp37 | 16 | Resistant | Italy | 1 | 512 |
| CCBH 22491 | 32 | Resistant | Brazil | 1 | 11 |
| CCBH 22240 | 32 | Resistant | Brazil | 1 | 48 |
| CCBH 22391 | 32 | Resistant | Brazil | 1 | 48 |
| CCBH 23171 | 32 | Resistant | Brazil | 4 | 111 |
| ATH10 | 32 | Resistant | Greece | 4 | 147 |
| CCBH 22999 | 32 | Resistant | Brazil | 4 | 147 |
| CCBH 23001 | 32 | Resistant | Brazil | 4 | 147 |
| 4_GR_12 | 32 | Resistant | Greece | 1 | 258 |
| ATH22 | 32 | Resistant | Greece | 1 | 258 |
| ATH30 | 32 | Resistant | Greece | 1 | 258 |
| CCBH 22143 | 32 | Resistant | Brazil | 1 | 258 |
| CCBH 22237 | 32 | Resistant | Brazil | 1 | 437 |
| CCBH 23024 | 32 | Resistant | Brazil | 1 | 437 |
| CCBH 23043 | 32 | Resistant | Brazil | 1 | 437 |
| CCBH 23048 | 32 | Resistant | Brazil | 1 | 437 |
| CCBH 23050 | 32 | Resistant | Brazil | 1 | 437 |
| CCBH 23247 | 32 | Resistant | Brazil | 1 | 437 |
| 12_BR_13 | 64 | Resistant | Brazil | 1 | 11 |
| CCBH 22206 | 64 | Resistant | Brazil | 1 | 11 |
| CCBH 22740 | 64 | Resistant | Brazil | 1 | 11 |
| CCBH 23296 | 64 | Resistant | Brazil | 1 | 11 |
| CCBH 22399 | 64 | Resistant | Brazil | 1 | 15 |
| CCBH 22609 | 64 | Resistant | Brazil | 1 | 15 |
| CCBH 22397 | 64 | Resistant | Brazil | 4 | 16 |
| CCBH 22675 | 64 | Resistant | Brazil | 4 | 16 |
| CCBH 23097 | 64 | Resistant | Brazil | 4 | 16 |
| CCBH 23650 | 64 | Resistant | Brazil | 4 | 17 |
| CCBH 22625 | 64 | Resistant | Brazil | 1 | 48 |
| CCBH 22128 | 64 | Resistant | Brazil | 1 | 76 |
| 7_GR_13 | 64 | Resistant | Greece | 1 | 258 |
| 8_GR_13 | 64 | Resistant | Greece | 1 | 258 |
| 10_GR_13 | 64 | Resistant | Greece | 1 | 258 |
| 18_GR_14 | 64 | Resistant | Greece | 1 | 258 |
| 19_GR_14 | 64 | Resistant | Greece | 1 | 258 |
| ATH24 | 64 | Resistant | Greece | 1 | 258 |
| ATH26 | 64 | Resistant | Greece | 1 | 258 |
| CCBH 22466 | 64 | Resistant | Brazil | 1 | 437 |
| CCBH 22481 | 64 | Resistant | Brazil | 1 | 437 |
| CCBH 23031 | 64 | Resistant | Brazil | 1 | 437 |
| CCBH 22653 | 128 | Resistant | Brazil | 1 | 11 |
| CCBH 23323 | 128 | Resistant | Brazil | 1 | 11 |
| CCBH 23661 | 128 | Resistant | Brazil | 4 | 147 |
| CCBH 23663 | 128 | Resistant | Brazil | 4 | 147 |
| ATH8 | 128 | Resistant | Greece | 1 | 258 |
| ATH16 | 128 | Resistant | Greece | 1 | 258 |
| ATH18 | 128 | Resistant | Greece | 1 | 258 |
| CCBH 23615 | 128 | Resistant | Brazil | 1 | 258 |
| CCBH 23741 | 128 | Resistant | Brazil | 1 | 340 |
| CCBH 23064 | 128 | Resistant | Brazil | 1 | 437 |
| CCBH 23220 | 128 | Resistant | Brazil | 27 | 3228 |
