# Supplementary material for: Genetic Diversity of Polymyxin Resistance Genes in Klebsiella pneumoniae Clinical Isolates
Source: Mol Ecol. 2026 Jan 20;35(2):e70234. doi: 10.1111/mec.70234 (PMC12817155; doi:10.1111/mec.70234)
Supplement: Supplementary file 4 — Table S3: mec70234‐sup‐0004‐TableS3.docx. [file MEC-35-e70234-s003.docx]

| **Table S3**: Aminoacid substitutions and MIC | | |  |  |  |  |  |  |  |
| --- | --- | --- | --- | --- | --- | --- | --- | --- | --- |
| **Strain** | **Haplotype** | ***mgrB*** | ***phoP*** | ***phoQ*** | ***pmrA*** | ***pmrB*** | **Alleles (N)** | **R/S** | **MIC** |
| ATH15 | 3 |  |  | N253T |  |  | 3 | S | 0,125 |
| ATH17 | 3 |  |  | N253T |  |  | 3 | S | 0,25 |
| Kp1001 | 0 |  |  |  |  |  | 80 | S | 0,25 |
| Kp1003 | 0 |  |  |  |  |  | 80 | S | 0,25 |
| Kp1019 | 0 |  |  |  |  |  | 80 | S | 0,25 |
| Kp1031 | 0 |  |  |  |  |  | 80 | S | 0,25 |
| Kp1032 | 0 |  |  |  |  |  | 80 | S | 0,25 |
| Kp1036 | 0 |  |  |  |  |  | 80 | S | 0,25 |
| Kp1122 | 0 |  |  |  |  |  | 80 | S | 0,25 |
| Kp1144 | 0 |  |  |  |  |  | 80 | S | 0,25 |
| Kp1209 | 0 |  |  |  |  |  | 80 | S | 0,25 |
| Kp1363 | 0 |  |  |  |  |  | 80 | S | 0,25 |
| Kp1495 | 0 |  |  |  |  |  | 80 | S | 0,25 |
| Kp1495 | 4 |  |  |  |  | L213M | 2 | S | 0,25 |
| Kp1528 | 5 |  |  |  | M66I |  | 1 | S | 0,25 |
| Kp1675 | 25 |  | S72L | S409R. H410Y |  | V280L | 1 | S | 0,25 |
| Kp2162 | 6 |  |  |  | E57G |  | 12 | S | 0,25 |
| Kp2209 | 0 |  |  |  |  |  | 80 | S | 0,25 |
| Kp2958 | 0 |  |  |  |  |  | 80 | S | 0,25 |
| Kp3270 | 0 |  |  |  |  |  | 80 | S | 0,25 |
| Kp3725 | 0 |  |  |  |  |  | 80 | S | 0,25 |
| Kp3860 | 2 |  |  |  | A41Y | T240M, L213M | 5 | S | 0,25 |
| Kp4194 | 0 |  |  |  |  |  | 80 | S | 0,25 |
| Kp4197 | 0 |  |  |  |  |  | 80 | S | 0,25 |
| Kp4256 | 0 |  |  |  |  |  | 80 | S | 0,25 |
| Kp4279 | 7 |  |  |  |  | M175V | 3 | S | 0,25 |
| Kp4287 | 0 |  |  |  |  |  | 80 | S | 0,25 |
| Kp4292 | 0 |  |  |  |  |  | 80 | S | 0,25 |
| Kp4333 | 0 |  |  |  |  |  | 80 | S | 0,25 |
| Kp4367 | 0 |  |  |  |  |  | 80 | S | 0,25 |
| Kp4387 | 0 |  |  |  |  |  | 80 | S | 0,25 |
| Kp4408 | 0 |  |  |  |  |  | 80 | S | 0,25 |
| Kp684 | 0 |  |  |  |  |  | 80 | S | 0,25 |
| Kp689 | 0 |  |  |  |  |  | 80 | S | 0,25 |
| Kp748 | 0 |  |  |  |  |  | 80 | S | 0,25 |
| Kp776 | 0 |  |  |  |  |  | 80 | S | 0,25 |
| Kp804 | 0 |  |  |  |  |  | 80 | S | 0,25 |
| Kp828 | 0 |  |  |  |  |  | 80 | S | 0,25 |
| Kp829 | 0 |  |  |  |  |  | 80 | S | 0,25 |
| Kp850 | 0 |  |  |  |  |  | 80 | S | 0,25 |
| Kp874 | 0 |  |  |  |  |  | 80 | S | 0,25 |
| Kp875 | 0 |  |  |  |  |  | 80 | S | 0,25 |
| Kp888 | 0 |  |  |  |  |  | 80 | S | 0,25 |
| Kp898 | 0 |  |  |  |  |  | 80 | S | 0,25 |
| Kp910 | 0 |  |  |  |  |  | 80 | S | 0,25 |
| Kp972 | 0 |  |  |  |  |  | 80 | S | 0,25 |
| Kp986 | 0 |  |  |  |  |  | 80 | S | 0,25 |
| Kp997 | 0 |  |  |  |  |  | 80 | S | 0,25 |
| 249 | 0 |  |  |  |  |  | 80 | S | 0,5 |
| AR_0363 | 16 |  |  | N255I, S350Y |  |  | 1 | S | 0,5 |
| ATH21 | 3 |  |  | N253T |  |  | 3 | S | 0,5 |
| ATH23 | 10 |  |  | V446G |  |  | 1 | S | 0,5 |
| ATH25 | 19 |  |  | L239P |  |  | 1 | S | 0,5 |
| ATH9 | 1 |  |  |  |  | T140P | 2 | S | 0,5 |
| CBAS 537 | 70 |  |  | D150G |  | P344L | 1 | S | 0,5 |
| Kp36 | 17 |  |  | S56R, I422S |  |  | 1 | S | 0,5 |
| Kp38 | 8 |  | A110S |  |  |  | 1 | S | 0,5 |
| Kp4246 | 0 |  |  |  |  |  | 80 | S | 0,5 |
| Kp4265 | 4 |  |  |  |  | L213M | 2 | S | 0,5 |
| kp46 | 9 |  |  | S56R |  |  | 2 | S | 0,5 |
| kp49 | 9 |  |  | S56R |  |  | 2 | S | 0,5 |
| 7585 | 0 |  |  |  |  |  | 80 | S | 1 |
| 7610 I | 72 |  |  |  |  | P346Q | 1 | S | 1 |
| CBAS 541 | 71 |  |  |  | E35A, M66I |  | 1 | S | 1 |
| JC 22 | 0 |  |  |  |  |  | 80 | S | 1 |
| JC 31 | 68 |  |  |  |  | T240M | 1 | S | 1 |
| Kp1507 | 0 |  |  |  |  |  | 80 | S | 1 |
| Kp1938 | 0 |  |  |  |  |  | 80 | S | 1 |
| Kp1990 | 0 |  |  |  |  |  | 80 | S | 1 |
| Kp2200 | 6 |  |  |  | E57G |  | 12 | S | 1 |
| Kp2224 | 0 |  |  |  |  |  | 80 | S | 1 |
| Kp2287 | 0 |  |  |  |  |  | 80 | S | 1 |
| Kp2334 | 6 |  |  |  | E57G |  | 12 | S | 1 |
| Kp2447 | 0 |  |  |  |  |  | 80 | S | 1 |
| Kp2454 | 0 |  |  |  |  |  | 80 | S | 1 |
| Kp2463 | 18 |  |  |  |  | T240M, V257A | 1 | S | 1 |
| Kp2476 | 7 |  |  |  |  | M175V | 3 | S | 1 |
| Kp2497 | 12 |  |  |  |  | A282S | 1 | S | 1 |
| Kp2564 | 0 |  |  |  |  |  | 80 | S | 1 |
| Kp2568 | 6 |  |  |  | E57G |  | 12 | S | 1 |
| Kp2587 | 6 |  |  |  | E57G |  | 12 | S | 1 |
| Kp2605 | 0 |  |  |  |  |  | 80 | S | 1 |
| Kp2606 | 6 |  |  |  | E57G |  | 12 | S | 1 |
| Kp2645 | 7 |  |  |  |  | M175V | 3 | S | 1 |
| Kp2786 | 0 |  |  |  |  |  | 80 | S | 1 |
| Kp2864 | 0 |  |  |  |  |  | 80 | S | 1 |
| Kp2895 | 0 |  |  |  |  |  | 80 | S | 1 |
| Kp2948 | 0 |  |  |  |  |  | 80 | S | 1 |
| Kp3000 | 14 |  |  | L28F |  |  | 1 | S | 1 |
| Kp3046 | 0 |  |  |  |  |  | 80 | S | 1 |
| Kp3185 | 0 |  |  |  |  |  | 80 | S | 1 |
| Kp3323 | 0 |  |  |  |  |  | 80 | S | 1 |
| Kp3509 | 0 |  |  |  |  |  | 80 | S | 1 |
| Kp3660 | 0 |  |  |  |  |  | 80 | S | 1 |
| Kp4195 | 0 |  |  |  |  |  | 80 | S | 1 |
| Kp4855 | 0 |  |  |  |  |  | 80 | S | 1 |
| Kp4856 | 11 |  |  |  | G53S |  | 1 | S | 1 |
| Kp4857 | 0 |  |  |  |  |  | 80 | S | 1 |
| Kp4862 | 0 |  |  |  |  |  | 80 | S | 1 |
| Kp4864 | 0 |  |  |  |  |  | 80 | S | 1 |
| Kp4865 | 0 |  |  |  |  |  | 80 | S | 1 |
| Kp4869 | 0 |  |  |  |  |  | 80 | S | 1 |
| Kp4871 | 2 |  |  |  | A41Y | T240M, L213M | 5 | S | 1 |
| Kp4878 | 2 |  |  |  | A41Y | T240M, L213M | 5 | S | 1 |
| Kp4886 | 0 |  |  |  |  |  | 80 | S | 1 |
| Kp4887 | 0 |  |  |  |  |  | 80 | S | 1 |
| Kp840 | 13 |  |  |  | I127V |  | 1 | S | 1 |
| Kp918 | 0 |  |  |  |  |  | 80 | S | 1 |
| Kp919 | 0 |  |  |  |  |  | 80 | S | 1 |
| 37 | 6 |  |  |  | E57G |  | 12 | S | 2 |
| 71 | 15 |  | D135N |  |  |  | 1 | S | 2 |
| CCBH33272 | 0 |  |  |  |  |  | 80 | S | 2 |
| CCBH72480 | 6 |  |  |  | E57G |  | 12 | S | 2 |
| CCBH73566 | 6 |  |  |  | E57G |  | 12 | S | 2 |
| CCBH73965 | 6 |  |  |  | E57G |  | 12 | S | 2 |
| CBAS 540 | 0 |  |  |  |  |  | 80 | S | 2 |
| JC 23 | 67 |  |  |  |  | E7Q | 1 | S | 2 |
| Kp1924 | 6 |  |  |  | E57G |  | 12 | S | 2 |
| Kp3851 | 0 |  |  |  |  |  | 80 | S | 2 |
| Kp4860 | 2 |  |  |  | A41Y | T240M, L213M | 5 | S | 2 |
| Kp4861 | 6 |  |  |  | E57G |  | 12 | S | 2 |
| Kp4882 | 0 |  |  |  |  |  | 80 | S | 2 |
| CCBH22404 | 58 |  |  |  | E57G | H61Q | 1 | R | 4 |
| CCBH23368 | 47 | K3T |  |  |  |  | 1 | R | 4 |
| AR_0125 | 26 | W20R |  |  |  |  | 1 | R | 4 |
| 201 | 69 | Insertion |  |  | L132M |  | 1 | R | 8 |
| 23_GR_12 | 28 | Insertion | A95S | V446G |  |  | 1 | R | 8 |
| AR_0454 | 27 | Insertion |  |  |  |  | 31 | R | 8 |
| 153 | 27 | Insertion |  |  |  |  | 31 | R | 16 |
| 154 | 27 | Insertion |  |  |  |  | 31 | R | 16 |
| 155 | 27 | Insertion |  |  |  |  | 31 | R | 16 |
| 219 | 27 | Insertion |  |  |  |  | 31 | R | 16 |
| 7559 | 0 |  |  |  |  |  | 80 | R | 16 |
| CCBH22137 | 27 | Insertion |  |  |  |  | 31 | R | 16 |
| CCBH22408 | 59 |  |  | Q405A |  |  | 1 | R | 16 |
| CCBH22462 | 50 | S36R |  |  |  |  | 2 | R | 16 |
| CCBH22997 | 38 | Insertion |  | V27H, F398K |  |  | 1 | R | 16 |
| CCBH23000 | 37 | Insertion |  | R16C |  | A282R | 1 | R | 16 |
| CCBH23167 | 63 |  |  | C395V | A41T | L213M | 1 | R | 16 |
| CCBH23454 | 66 |  |  |  |  | V280L | 1 | R | 16 |
| 13_GR_14 | 29 | Insertion |  |  |  | P158R | 1 | R | 16 |
| 9_GR_12 | 20 |  |  | T281M |  |  | 1 | R | 16 |
| Kp19 | 27 | Insertion |  |  |  |  | 31 | R | 16 |
| Kp34 | 27 | Insertion |  |  |  |  | 31 | R | 16 |
| Kp37 | 27 | Insertion |  |  |  |  | 31 | R | 16 |
| Kp4164 | 24 |  |  | H339D | E57G |  | 1 | R | 16 |
| Kp4889 | 2 |  |  |  | A41Y | T240M, L213M | 5 | R | 16 |
| Kp5505 | 49 | Q30Stop |  |  |  | M175V | 1 | R | 16 |
| Kp5506 | 31 | Insertion |  |  | E57G |  | 2 | R | 16 |
| Kp5508 | 23 |  |  |  |  | T157P, M175V | 1 | R | 16 |
| Kp5509 | 46 | K3Stop |  |  | E57G |  | 1 | R | 16 |
| Kp5510 | 30 | Insertion |  |  |  | M175V | 4 | R | 16 |
| Kp5511 | 31 | Insertion |  |  | E57G |  | 2 | R | 16 |
| Kp5513 | 30 | Insertion |  |  |  | M175V | 4 | R | 16 |
| Kp5514 | 30 | Insertion |  |  |  | M175V | 4 | R | 16 |
| Kp5516 | 30 | Insertion |  |  |  | M175V | 4 | R | 16 |
| Kp5520 | 22 |  |  |  |  | M175V, G207D | 1 | R | 16 |
| 129 | 45 | K3STOP |  |  |  |  | 4 | R | 32 |
| CCBH22143 | 55 |  |  | I88N |  |  | 1 | R | 32 |
| CCBH22237 | 27 | Insertion |  |  |  |  | 31 | R | 32 |
| CCBH22240 | 27 | Insertion |  |  |  |  | 31 | R | 32 |
| CCBH22391 | 27 | Insertion |  |  |  |  | 31 | R | 32 |
| CCBH22491 | 27 | Insertion |  |  |  |  | 31 | R | 32 |
| CCBH22999 | 32 | Insertion |  |  |  | A282R | 1 | R | 32 |
| CCBH23001 | 27 | Insertion |  |  |  |  | 31 | R | 32 |
| CCBH23024 | 41 | Insertion |  | V27H, P103W, C395A |  |  | 2 | R | 32 |
| CCBH23043 | 53 | S36R |  | V27H, P103W, C395A |  |  | 1 | R | 32 |
| CCBH23048 | 51 | S36R |  |  |  | A282R | 1 | R | 32 |
| CCBH23050 | 52 | S36R |  | P103W, C395A |  |  | 1 | R | 32 |
| CCBH23171 | 45 | K3STOP |  |  |  |  | 4 | R | 32 |
| CCBH23247 | 33 | Insertion |  |  |  | L213M | 1 | R | 32 |
| 4_GR_12 | 39 | Insertion | P74L | N253T |  |  | 1 | R | 32 |
| ATH10 | 1 |  |  |  |  | T140P | 2 | R | 32 |
| ATH22 | 34 | Insertion |  | N253P |  |  | 2 | R | 32 |
| ATH30 | 35 | Insertion |  | V446G |  |  | 1 | R | 32 |
| 257 | 0 |  |  |  |  |  | 80 | R | 64 |
| 417 | 45 | K3STOP |  |  |  |  | 4 | R | 64 |
| 446 | 45 | K3STOP |  |  |  |  | 4 | R | 64 |
| CCBH22128 | 54 |  | G121A |  |  |  | 1 | R | 64 |
| CCBH22206 | 41 | Insertion |  | V27H, P103W, C395A |  |  | 2 | R | 64 |
| CCBH22382 | 27 | Insertion |  |  |  |  | 31 | R | 64 |
| CCBH22397 | 56 |  |  | V27H, Y265C | E57G |  | 1 | R | 64 |
| CCBH22399 | 57 |  |  | T246C |  |  | 1 | R | 64 |
| CCBH22466 | 50 | S36R |  |  |  |  | 2 | R | 64 |
| CCBH22481 | 27 | Insertion |  |  |  |  | 31 | R | 64 |
| CCBH22609 | 60 |  |  | V27H, Y265T |  |  | 1 | R | 64 |
| CCBH22625 | 27 | Insertion |  |  |  |  | 31 | R | 64 |
| CCBH22675 | 61 |  |  | Y265C |  |  | 1 | R | 64 |
| CCBH22740 | 62 |  |  | E397G |  |  | 1 | R | 64 |
| CCBH23031 | 43 | Insertion |  | V27H, P103W, C395A |  | A282R | 1 | R | 64 |
| CCBH23097 | 44 | Insertion |  | R16A, V27H, D73I, P103W, S188T | R160S, E57G |  | 1 | R | 64 |
| CCBH23296 | 27 | Insertion |  |  |  |  | 31 | R | 64 |
| CCBH23650 | 48 | L8STOP |  |  | E57G | G345R | 1 | R | 64 |
| 10_GR_13 | 42 | Insertion |  | G39S, A225T, N253T |  |  | 1 | R | 64 |
| 12_BR_13 | 27 | Insertion |  |  |  |  | 31 | R | 64 |
| 18_GR_14 | 27 | Insertion |  |  |  |  | 31 | R | 64 |
| 19_GR_14 | 27 | Insertion |  |  |  |  | 31 | R | 64 |
| 7_GR_13 | 27 | Insertion |  |  |  |  | 31 | R | 64 |
| 8_GR_13 | 21 |  |  | G385C |  |  | 1 | R | 64 |
| ATH10 | 27 | Insertion |  |  |  |  | 31 | R | 64 |
| ATH24 | 27 | Insertion |  |  |  |  | 31 | R | 64 |
| ATH26 | 27 | Insertion |  |  |  |  | 31 | R | 64 |
| CCBH22653 | 36 | Insertion |  |  |  | L222A | 1 | R | 128 |
| CCBH23064 | 27 | Insertion |  |  |  |  | 31 | R | 128 |
| CCBH23220 | 64 |  |  |  |  | M285L | 1 | R | 128 |
| CCBH23323 | 65 |  |  |  | L63H |  | 1 | R | 128 |
| CCBH23615 | 27 | Insertion |  |  |  |  | 31 | R | 128 |
| CCBH23661 | 27 | Insertion |  |  |  |  | 31 | R | 128 |
| CCBH23663 | 27 | Insertion |  |  |  |  | 31 | R | 128 |
| CCBH23741 | 27 | Insertion |  |  |  |  | 31 | R | 128 |
| ATH16 | 40 | Insertion |  | N253T, T439P |  |  | 1 | R | 128 |
| ATH18 | 34 | Insertion |  | N253P |  |  | 2 | R | 128 |
| ATH8 | 27 | Insertion |  |  |  |  | 31 | R | 128 |
